# Productivity, real wages, and gender. A study in Colombian manufacturing

**DOI:** 10.12688/f1000research.161343.1

**Published:** 2025-03-17

**Authors:** Andrés Mauricio Gómez-Sánchez, Zoraida Ramírez-Gutiérrez, Isabel Cristina Rivera-Lozada

**Affiliations:** 1 Departament of Economics, Universidad del Cauca, Popayan, Cauca, Colombia; 2 Departament of Accounting, Universidad del Cauca, Popayan, Cauca, Colombia; 3 Departament of Economics, Universidad del Cauca, Popayan, Cauca, Colombia

**Keywords:** Wages, Gender, Firms productivity, Manufacturing, Emerging Economy

## Abstract

**Background:**

Orthodox microeconomic theory establishes a positive link between employee wages and productivity in competitive markets. However, this perspective often overlooks gender and treats the workforce as a homogeneous category labelled as labor, potentially obscuring issues of gender discrimination. This article addresses the gender wage gap by analyzing, for the first time, at least in an emerging economy such as Colombia, the impact of manufacturing firm’s productivity on wages explicitly including gender.

**Method:**

First, we use the GMM two-equation system proposed by Wooldridge (2009) to obtain consistent and unbiased estimates of output elasticities and TFP, respectively. Secondly, we explore wages-productivity linkage by gender, implementing a dynamic random effect generalized least squares model (GLS) with panel data to deal with endogeneity issues.

**Results:**

Our main findings reveal, among others, that firms with a higher proportion of female workers (female firms) generally have higher productivity than those with a higher proportion of male workers (male firms).

**Conclusions:**

The effect of female firm’s productivity on wages is lower than that of male firm’s productivity, which could indicate gender wage discrimination

## Introduction

 The economic literature, particularly within the industrial organization, suggests that firm wages depend on several factors, including firm size ( Carlsson, et al. 2016), employee education and skills ( Mincer, 1974; Schultz, 1961), economic subsector ( Suhányi, et al. 2023), geographic location ( Méjean & Patureau, 2010), and the presence or absence of unions ( Card, et al. 2017). Nevertheless, productivity is the most critical factor influencing wages ( Feldstein, 2008).

Based on the neoclassical microeconomic theory, increases in labor productivity positively affect real wage growth in the long-run ( Mankiw, 2015). In line with this, industrial organization literature acknowledges that firm productivity significantly influences employees’ wages. Although empirical evidence supporting this hypothesis varies in strength depending on the country and industries analyzed, productivity is widely recognized as a key determinant of wages, whether at the individual or firm level ( Meager & Speckesser, 2011).

Despite this, previous literature has left two issues aside. First, the method used to measure productivity and second, the role of gender in these topics. Regarding productivity measurement, several studies, particularly in macroeconomics, have used the average product of labor as a proxy. This approach is limited as it measures labor productivity rather than firm productivity and only applies in the short-run ( Sargent & Rodriguez, 2000). While other studies, particularly in microeconomics, have correctly used total factor productivity (TFP) at the firm level, many studies generate flawed estimates due to the use of statistical methods that generate biased and inconsistent parameters, leading to inaccurate conclusions and recommendations ( Gómez Sánchez, 2020).

The second issue is the exclusion of gender from productivity analysis. Microeconomic and Industrial Organisation theory often treats employees under the label “labor.”, which can lead to biases, especially in emerging economies such as Colombia, where industrial subsectors such as Beverages, Garments, Textiles, Pharmaceuticals, or Chemicals, a significant proportion of employees are female. This is an important consideration, as productivity may respond differently to the presence of women than men, and this difference should influence employees' wages.

In this order of ideas, this paper aims to assess the relationship between wages and productivity in firms with a high proportion of female employees or *female firms* hereafter, compared to firms with a higher proportion of male employees, referred to as *male firms* hereafter ( Tsou & Yang, 2019; Gomez Sanchez, et al., 2025). To do so, we first estimate unbiased total factor productivity using the two-stage method proposed by Wooldridge (2009), and for the first time, we disaggregate the workforce into male and female to obtain gender- a gender-specific productivity estimate rather than an aggregate measure of TFP as the tradition.

In this regard, we implement the methodology in two steps. First, we use the two-equation system proposed by Wooldridge (2009) to obtain consistent estimates of input elasticities and unbiased TFP estimates. This system is jointly estimated under the Generalised Method of Moments (GMM) framework. As a novelty, we separate employees by gender to estimate TFP. Second, we implement a dynamic random effect generalized least squares model (GLS) with panel data to address endogeneity. Moreover, we also include industrial organization covariates, such as exports or innovations, along with wage persistence; that is, firms with higher (or lower) wages in the past tend to continue those wage levels in the present. In addition, the model also deals with potential initial condition problems ( Blundell & Bond, 1998).

The data comes from The Annual Manufacturing Survey (EAM) and The Technological Development and Innovation Survey (EDIT), both published by the Statistics Department of Colombia (DANE) for the period 2013-2020. After merging eight waves of EAM and EDIT, we obtain an unbalanced panel with 59,355 observations. Our main findings are summarised as follows: i) Female firms exhibit higher productivity than male firms. ii) The impact of female firms’ productivity on wages is lower than female firms’ productivity, suggesting wage discrimination in the Colombian manufacturing industry. iii) Female labor output elasticity is higher than male elasticity, indicating that women contribute more to firm output than men iv) TFP displays lower input elasticities than gender-specific TFP estimates, suggesting that TFP underestimates firm productivity when gender is considered.

The remainder of this paper is organized as follows. Section 2 examines theoretical and empirical literature. Section 3 describes the data. Section 4 displays TFP and stochastic model estimates. Section 5 discusses the results, and Section 6 offers conclusions.

## Methods

### Literature review

The existing literature on gender and productivity pay disparities primarily focuses on led-women or led-male firms. These definitions differ slightly from the concept of male and female firms explored in this article. Due to data limitations in EAM survey, we classify firms as *female* or *male* based on the proportion of female or male workers employed, respectively, following the approach of Tsou and Yang (2019). Consequently, much of the literature is less concerned with our adopted firm’s definition, which focuses on led-women or led-male firms.

The orthodox microeconomic theory postulates that a worker's productivity positively correlates with wages, as outlined in the efficiency wage theory ( Akerlof & Yellen, 1986; Shapiro & Stiglitz, 1984). Nevertheless, this paper demonstrates that wages do not necessarily align with a firm’s productivity. It highlights how female-led firms often exhibit higher productivity than male-led ones, yet wage disparities do not reflect these productivity differences. This phenomenon can be attributed to gender wage gaps masked by the low levels of gender parity worldwide ( World Economic Forum, 2024) and employers’ perceptions of female worker's productivity being negatively influenced by traditional caregiving roles and motherhood responsibilities ( Todaro et al., 2002).

As Goldin (2014) argues, women have adapted their participation in the labor market over time. Initially, they balanced household and work life, often leaving their employments during motherhood. Subsequently, they increased their educational attainment, equalling or even surpassing that of men to remain competitive in the workforce. Despite these efforts, wage compensation has not reflected women’s labor market resilience. Gender wage gaps persist globally ( World Bank, 2024), and wage disparities widen with age.

In Colombia, since the early days of industrialization, women have played a significant role in the workforce, particularly in sectors like Food and Textiles. This participation was largely driven by the need to supplement family income ( Santos, 2017; Arango, 1991). Nonetheless, their contributions were often associated with low wages, influenced by religiously rooted work ethics and disciplinary norms ( Arango, 1991). More recently, gender wage gaps have persisted despite evidence showing that female-led firms perform better. For instance, women-led firms, identified through the Unified Business and Social Registry (RUES, by its Spanish acronym), account for 59% of Colombia’s 1.2 million registered firms and 1.8 million microenterprises owned by women, according to Emicron Survey (2022) published by National Statistics Department. These firms report higher productivity (34.3% compared to 33.25% for men) and more significant efficiency improvements [25.8% for women versus 17.9% for men] ( WCP, 2024).

Santos (2017), considers that despite their higher productivity and significant contributions to the manufacturing sector, wage firms differentials in this country have consistently exceeded productivity gaps. This is theoretically grounded on three factors. Firstly, following Todaro et al., (2002), perceived costs of female labor as hired women are often considered more expensive due to factors related to motherhood, which are believed to affect productivity, despite firms lacking systems for measuring labor costs disaggregated by sex. Secondly, Santos (2017) suggests that wages diverge from efficiency theory: women-led firms take advantage of relatively lower wages to increase profits, contrary to efficiency wage theory. Thirdly, Goldin, (2014) pointed out the sectoral occupational segregation, that is, led-women firms tend to concentrate on specific sectors, perpetuating occupational segregation.

The scarce international literature on these topics shows mixed results. Tsou and Yang (2019) find that Chinese firms with a higher proportion of female workers generally have lower productivity than those with a higher proportion of male workers. However, female workers with a high education level significantly improve the firm’s productivity, especially in small private and foreign firms, but not for medium or large public ones. These contradictory results also depend on the statistical method used. Pfeifer and Wagner (2014) find lower productivity in female firms than in male firms under the OLS method, whilst they obtain opposite results if they use a GMM framework.

### TFP estimates by gender

TFP broken down by gender follows Wooldridge’s (2009) method. This framework takes advantage of the GMM estimation to shorten the standard error calculation and avoid using bootstrapping techniques. Expressly, we assume that the production process follows a linearised Cobb-Douglas production function:

$$y_{\mathrm{it}}=\beta_{0}+\beta_{\mathrm{lw}}l_{\mathrm{it}}^{w}+\beta_{\mathrm{lm}}l_{\mathrm{it}}^{m}+\beta_{k}k_{\mathrm{it}}+\beta_{m}m_{\mathrm{it}}+\beta_{e}{\mathrm{ene}}_{\mathrm{it}}+\omega_{\mathrm{it}}+\varepsilon_{\mathrm{it}}$$ (1)

$y_{\mathrm{it}}$ denotes firm’s *i* production in period *t*; $l_{\mathrm{it}}^{w}$ is the women labor;; $l_{\mathrm{it}}^{m}$ is the men labor; $k_{\mathrm{it}}$ is the firm’s capital stock; and, $m_{\mathrm{it}}$ is the materials consumption, and ${\mathrm{ene}}_{\mathrm{it}}$ is the electric energy consumption. Besides, $\omega_{\mathrm{it}}$ is the firm’s productivity, which is only observable or predictable by the firm. As well, $\varepsilon_{\mathrm{it}}$ represents an error term unobserved or unpredictable by firms.

Olley & Pakes (1992) assume that capital stock evolves according to the perpetual inventory method, and it is determined in the previous period (state variable). Therefore, current productivity shocks do not affect capital stock ( Eslava et al., 2013). We also assume that labor by gender and firm’s energy consumption are chosen in the same period, as they are consumed (freely variable factors). However, Ackerberg, et al., (2015) demonstrate that these choices are correlated with $\;\omega_{\mathrm{it}}$ , so the equation (1) cannot be estimated by ordinary least squares (OLS), fixed effects (FE), or instrumental variables (IV). In this sense, Levinsohn and Petrin (2003) suggests using the materials demand function as a proxy for unobserved productivity. The demand for materials is as follows:

$$m_{\mathrm{it}}=m_{t}\left(k_{\mathrm{it}},\omega_{\mathrm{it}},{\mathrm{ene}}_{\mathrm{it}}\right)$$ (2)

According to Levinsohn and Petrin (2003), this demand is monotonically increasing in productivity, so we can invert this function to express firms’ productivity in terms of observables:

$$\omega_{\mathrm{it}}=m_{t}^{-1}\left(k_{\mathrm{it}},\omega_{\mathrm{it}},{\mathrm{ene}}_{\mathrm{it}}\right)=h_{t}\left(k_{\mathrm{it}},\omega_{\mathrm{it}},{\mathrm{ene}}_{\mathrm{it}}\right)$$ (3)

$h_{t}$ is an unknown function of $k_{\mathrm{it}}$ ; $m_{\mathrm{it}};k_{\mathrm{it}};\omega_{\mathrm{it}};$ and $\;{\mathrm{ene}}_{\mathrm{it}}$ . Replacing (3) in (1):

$$y_{\mathrm{it}}=\beta_{0}+\beta_{\mathrm{lw}}l_{\mathrm{it}}^{w}+\beta_{\mathrm{lm}}l_{\mathrm{it}}^{m}+\beta_{k}k_{\mathrm{it}}+\beta_{m}m_{\mathrm{it}}+\beta_{e}{\mathrm{ene}}_{\mathrm{it}}+h_{t}\left(k_{\mathrm{it}},\omega_{\mathrm{it}},{\mathrm{ene}}_{\mathrm{it}}\right)+\varepsilon_{\mathrm{it}}$$ (4)

$h_{t}\left(∙\right)\;$ is unknown, so it is proxied by third-degree polynomials in the respective arguments. Nevertheless, $\beta_{m}$ and $\beta_{k}$ are collinear with $h_{t}\left(∙\right),$ so we cannot to identify these parameters. Following Olley & Pakes (1992), and Levinsohn and Petrin (2003), it is necessary to introduce the law of motion of productivity as a Markov process:

$$\omega_{\mathrm{it}}=E\left[\omega_{\mathrm{it}}|\omega_{\mathrm{it}-1}\right]+\xi_{\mathrm{it}}=f\left(\omega_{\mathrm{it}-1}\right)+\xi_{\mathrm{it}}$$ (5)

where f(∙) is an unknown function that join productivity in t and in t−1 plus an innovation term, $\xi_{\mathrm{it}},$ which is uncorrelated to $k_{\mathrm{it}}$ . By replacing (5) in (1), we get:

$$y_{\mathrm{it}}=\beta_{0}+\beta_{l}l_{\mathrm{it}}+\beta_{k}k_{\mathrm{it}}+\beta_{m}m_{\mathrm{it}}+\beta_{e}{\mathrm{ene}}_{\mathrm{it}}+\beta_{w}{\mathrm{wat}}_{\mathrm{it}}+f\left(\omega_{\mathrm{it}-1}\right)+\xi_{\mathrm{it}}+\varepsilon_{\mathrm{it}}$$ (6)

Furthermore, by lagging and replacing equation (3) into (6), we get:

$$y_{\mathrm{it}}=\beta_{0}+\beta_{\mathrm{lw}}l_{\mathrm{it}}^{w}+\beta_{\mathrm{lm}}l_{\mathrm{it}}^{m}+\beta_{k}k_{\mathrm{it}}+\beta_{m}m_{\mathrm{it}}+\beta_{e}{\mathrm{ene}}_{\mathrm{it}}+h\left(k_{\mathrm{it}-1},m_{\mathrm{it}-1},{\mathrm{ene}}_{\mathrm{it}-1}\right)+v_{\mathrm{it}}$$ (7)

where $v_{\mathrm{it}}\;$ is a composed error term ( $\xi_{\mathrm{it}}+\varepsilon_{\mathrm{it}})$ . Because h(∙) is an unknown function, it is proxied by third degree polynomials in the arguments. Equations (4) and (7) are the two-equation systems proposed by Wooldridge (2009); they are jointly estimated by using the GMM method with suitable instruments and moment conditions showed for instance by Gómez Sánchez (2020). Once we estimate equation (1), we get the TFP as a residual by using output elasticities:

$$y_{\mathrm{it}}-\left({\hat{\beta }}_{0}+{\hat{\beta }}_{\mathrm{lw}}l_{\mathrm{it}}^{w}+{\hat{\beta }}_{\mathrm{lm}}l_{\mathrm{it}}^{m}+{\hat{\beta }}_{k}k_{\mathrm{it}}+{\hat{\beta }}_{m}m_{\mathrm{it}}+{\hat{\beta }}_{e}{\mathrm{ene}}_{\mathrm{it}}\right)={\hat{\omega }}_{\mathrm{it}}\;$$ (8)

where ${\hat{\omega }}_{\mathrm{it}}\;$ is the TFP estimated in logs for firm i at time t .

### Data analysis ^1^

This section describes some key variables used in this study that provide insight into the empirical model. We specifically examine the output elasticity of labor without considering gender and with gender taken into account, as well as the linkage with firms’ salaries, analyzed for the entire sample and by manufacturing sectors.

Table 1 presents the estimates of labor product elasticities using three different methods: Ordinary Least Squares (OLS), Generalized Least Squares (GLS), and Wooldridge’s (2009) two-step method. These estimates are analyzed under two scenarios: one considering the firm's total labor force (Labor) and the other distinguishing employees by gender (women and men’s labor).

**Table 1.** Estimates of labor output elasticities. Total labor and labor by gender.

	OLS	GLS	Wooldridge	OLS	GLS	Wooldridge
Labor	0.298***	0.252***	0.286***			
	(0.002)	(0.003)	(0.002)			
Women labor				0.141***	0.100***	0.136***
				(0.001)	(0.002)	(0.001)
Men labor				0.136***	0.137***	0.132***
				(0.002)	(0.003)	(0.002)
Capital	0.042***	0.023***	0.089***	0.045***	0.022***	0.093***
	(0.001)	(0.002)	(0.006)	(0.001)	(0.002)	(0.006)
Materials	0.711***	0.701***	0.684***	0.715***	0.705***	0.690***
	(0.001)	(0.002)	(0.003)	(0.002)	(0.002)	(0.003)
Energy	0.008***	0.056***	0.039***	0.016***	0.061***	0.045***
	(0.001)	(0.002)	(0.003)	(0.001)	(0.002)	(0.003)
Constant	3.096***	3.108***		3.222***	3.257***	
	(0.014)	(0.024)		(0.017	(0.026	
P-value Wald		0.000	0.000		0.000	0.000
P-value F	0.000			0.000		
Observations	67,781	67,781	55,624	65,879	65,879	53,799

Source: Author’s elaboration.

Robust standard error reported in parentheses.

***, ** and * indicate significance at 1, 5 and 10%, respectively.

As expected, the results generally reveal that all methods yield positive and statistically significant elasticities. Nevertheless, without distinguishing by gender, the product elasticity under Wooldridge’s (2009) method is 0.286. In contrast, the OLS and GLS methods tend to overestimate this figure, likely due to biases from unaddressed endogeneity issues in the Cobb-Douglas production function. When gender is considered, Wooldridge’s (2009) method shows that the product elasticity of women’s labor is 0.136, while that of men’s labor is 0.132. The remaining elasticities corresponding to capital (0.093), materials (0.690), and energy (0.045) fall within the ranges reported by empirical studies on the industrial organization in Colombia ( Gómez-Sánchez et al., 2022; Llopis et al., 2022; Sanchis Llopis et al., 2024).

The differential in labor product elasticities between men and women is noteworthy: the elasticity of female labor is higher, implying that women’s average contribution to the firms’ output surpasses that of men. However, it is possible that women’s wages do not correspond to this greater contribution to production. In this regard, we present additional descriptive analyses to explore this idea.

Figure 1 shows that male firms have higher average log-wages (13.52) than female firms (13.09). Besides, in terms of average product of labor (APL), male firms exhibit higher productivity (11.598) than female firms (11.127), seemingly aligning with the prediction of neoclassical theory.

**Figure 1.**
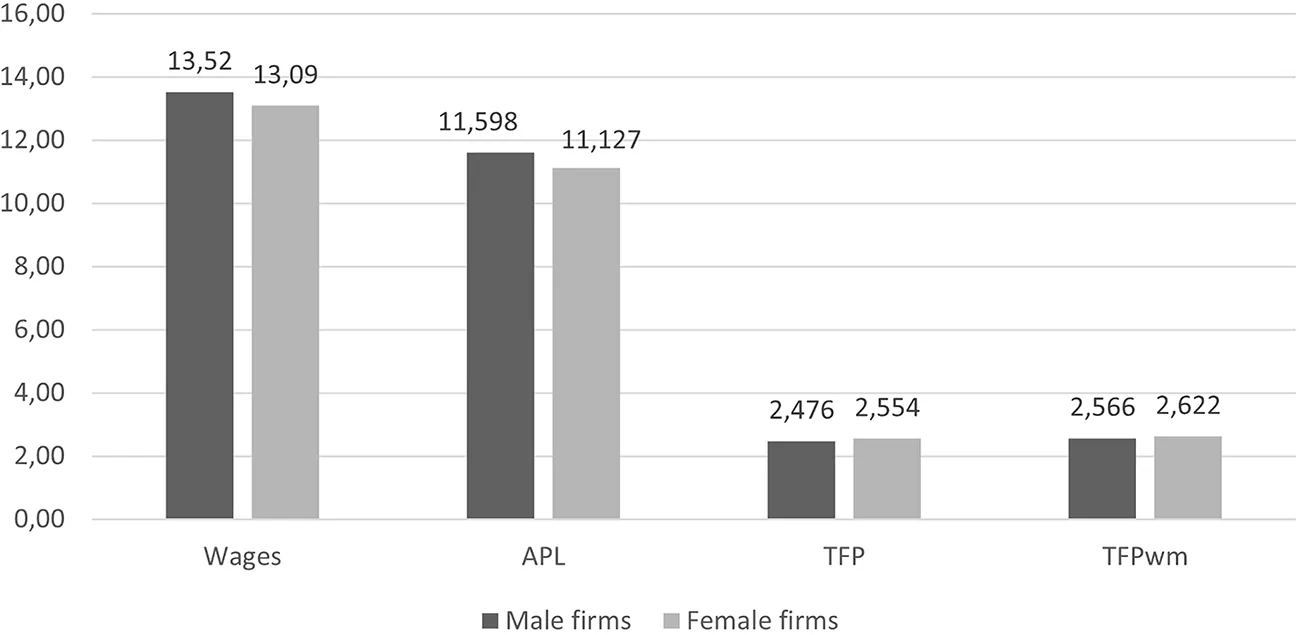
Wages and Productivity by female and male firms. Average in Logarithms.

Source: Author’s elaboration.

However, more precisely, measures of a firm’s productivity suggest different results. In the TFP scenario, female firms outperform male firms, with values of 2.554 and 2.476, respectively. Furthermore, in TFP weighted by gender (TFPwm), female firms also perform better (2.622) compared to male firms (2.566). Whilst further analysis is required to establish causality, these findings advise a potential productivity advantage when gender weighting is considered.

Table 2 presents wages and different productivity measures broken down by manufacturing sectors to deepen our analysis. In our preferred scenario (TFPwm), the numbers reveal that in industrial sectors such as Food, Textiles, Leather, Publishing, Coking, chemicals, Electric motors, Vehicles, and Machine Maintenance, female firms’ productivity displays higher productivity than male firms. However, wages are lower for female firms compared to salaries in male firms. In the remaining sectors, there is a clear correspondence between productivity and wages, that is, the more productive, the higher the wages, regardless of firms’ gender orientation.

**Table 2.** Wages and Productivity. Female and male firms by sector. Average in Logs.

Industry	Male Firms				Female Firms			
ISIC Rev.2	Wages	APL	TFP	TFPwm	Wages	APL	TFP	TFPwm
Food	13.874	12.089	2.437	2.501	12.901	11.179	2.452	2.511
Beverages	14.368	12.127	2.612	2.686	12.856	11.365	2.594	2.640
Textiles	13.874	11.427	2.346	2.416	13.036	11.078	2.493	2.554
Garment	12.809	11.585	2.591	2.637	13.092	10.979	2.625	2.727
Leather	12.739	11.168	2.424	2.495	12.724	10.939	2.487	2.547
Wood	12.838	11.263	2.427	2.554	12.132	10.875	2.398	2.450
Paper	14.310	12.128	2.412	2.486	12.982	11.187	2.418	2.463
Publishing	13.214	11.340	2.470	2.524	12.735	11.152	2.533	2.569
Coking	13.925	13.397	2.758	2.826	12.831	12.910	2.949	2.987
Chemical	13.888	12.273	2.615	2.682	13.665	11.466	2.686	2.750
Pharmaceutical	14.685	12.058	2.838	2.888	14.137	11.508	2.803	2.859
Rubber and Plastic	13.505	11.589	2.377	2.453	13.342	11.212	2.377	2.423
Non-Metallic Mineral Prod	13.823	11.554	2.384	2.522	12.784	11.019	2.404	2.439
Metallurgical Products	13.596	11.844	2.442	2.558	12.298	11.525	2.433	2.465
Metal products	13.178	11.204	2.509	2.630	12.552	10.941	2.472	2.520
Manufacture of Electronics	13.293	11.744	2.643	2.705	12.679	10.941	2.488	2.562
Electric motors	13.900	11.432	2.453	2.545	13.018	11.055	2.509	2.563
Machinery and Equipment	13.305	11.239	2.581	2.699	12.200	11.176	2.563	2.603
Vehicles	13.554	11.460	2.424	2.548	13.479	11.431	2.531	2.607
Ships and Boats	14.052	11.523	2.326	2.434	12.459	11.131	2.378	2.424
Furniture	13.065	11.090	2.456	2.558	12.249	11.078	2.447	2.489
Other manufactures	13.364	11.505	2.541	2.636	13.014	11.075	2.561	2.619
Machine Maintenance	13.596	11.673	2.645	2.772	11.966	11.226	2.792	2.830

Source: Author’s elaboration.

Other productivity measures, such as TFP, are consistent with the observed results for TFPwm. Nonetheless, APL estimates indicate that both productivity and wages are higher in male firms than in female firms across all industries except the Garment sector.

### Empirical model and estimates

To explore the effect of *female firm* productivity on a firm’s wages, we offer a dynamic generalized least squares model (GLS) with random effects for panel data. The variables are in natural logs except for dummy variables. In addition, we lagged all covariates in one period to deal with possible simultaneity, except factor variables such as time, industry, and firm localization. The model is as follows:

$${\mathrm{lw}}_{\mathrm{it}}=\varphi_{0}+\alpha_{0}{\mathrm{lw}}_{\mathrm{it}-1}+\alpha_{1}{\text{findx}}_{\mathrm{it}-1}+\alpha_{2}{\text{lprod}}_{\mathrm{it}-1}^{h,j,p}+\gamma_{k}Z_{\mathrm{it}-1}+\mathrm{pre}\bar{\mathrm{lw}}+{\mathrm{loc}}_{j}+{\text{year}}_{t}+{\mathrm{ind}}_{j}+\varepsilon_{\mathrm{it}}$$ (9)

Where ${\mathrm{lw}}_{\mathrm{it}}$ signifies the firms wages; $\;{\text{findx}}_{\mathrm{it}-1}^{q,r}$ represents the *female firm’s* index with *q* ∈ continuous index and *r* ∈ dichotomous index. Besides, ${\text{lprod}}_{\mathrm{it}}^{h,j,p}$ denotes firms productivity; with *h* ∈ full TFP; *j* ∈ TFP by gender; and *p* ∈ APL. Supported in Roberts and Tybout (1997), we introduce the term ${\text{lprod}}_{\mathrm{it}-1}^{h,j,p}$ to capture firm’s *wages persistence*, that is, firms with higher (lower) wages in the past; currently continue to show higher (lower) wages. In this sense, here persistence aims to capture potential gender discrimination. That is, it examines whether firms that paid lower salaries in female firms in the past continue to do so in the present.

The vector $Z_{\mathrm{it}-1}$ includes firm’s control variables according to industrial organisation. It includes the firm’s classification as SMEs (SMEs), to capture if a firm's size influences wages. In line with Schultz (1961), Mincer (1974), and others, we incorporate employee skills ( *skills*) as a proxy for human capital, as higher levels of education and/or training are associated with increased wages. Specifically, skills are the number of employees with master’s degrees. Export ( $\exp_{\mathrm{it}})\;$ and innovations activities $\;({\mathrm{inn}}_{\mathrm{it}}$ ) are included as a part of firm’s internationalisation strategies, as highlighted by De Loecker (2013) and Crepon, Duguet & Mairesse, (1998). According to Schank et al., (2010), firms with strong international trade connections and innovation efforts tend to be more productive and efficient, which typically results in higher employee wages. It is worth mentioning that due to the limited introduction of innovations in Colombian manufacturing, we combine both process and product innovations to achieve accurate statistical representativeness.

As well, the vector $Z_{\mathrm{it}-1}\;$ accounts for market concentration proxied by Herfindahl-Hirschman index ( *lihh*), and firm’s mark-up ( *lmarkup*). We also include the firms’ age ( *lage*) because, according to the ILO (2015), young small enterprises in Latin America significantly contribute to job creation. On the other hand, following Blundell and Bond (1998), we include pre-sample means of the dependent variable ( pre_lw2013 ) to deal with correlated unobserved firm heterogeneity in the model estimation. Note that we also control for geographic firm localization (loc), macroeconomic shocks ( *year*), and sector characteristics ( *ind*). Finally, $\varepsilon_{\mathrm{it}}$ is a composed the error term that consist of a fixed effect of firms ( $u_{i})$ and an idiosyncratic error term ( $\eta_{\mathrm{it}})$ .

## Results

Table 3 shows the estimates of the empirical modelling. Columns (1), (2), and (3) display TFP results for the full industry, female firms, and male firms, respectively. Columns (4), (5), and (6) present TFP results classified by workforce gender, whilst the final three columns analyze the average product of labor (APL). Each scenario includes continuous and dichotomous indices for *female firms* and wage *persistence.*

**Table 3.** Empirical Estimates.

		*TFP*			*TFPwm*			*APL*	
	*Industry*	*Female firm*	*Male firm*	*Industry*	*Female firm*	*Male firm*	*Industry*	*Female firm*	*Male firm*
*lrw*_*t*-1_	0.701**	0.587**	0.619**	0.668**	0.591**	0.592**	0.701**	0.587**	0.619**
	(0.010)	(0.019)	(0.012)	(0.010)	(0.020)	(0.012)	(0.010)	(0.019)	(0.012)
*ltfp*_*t*-1_	0.038**	0.055*	0.050**						
	(0.015)	(0.031)	(0.019)						
${\mathrm{ltfp}}_{t-1}^{\mathrm{wm}}$				0.059**	0.057*	0.062**			
				(0.015)	(0.031)	(0.019)			
*lapl*_*t*−1_							0.016**	0.021*	0.021**
							(0.005)	(0.010)	(0.006)
*smes*_*t*−1_	-0.476**	-0.535**	-0.574**	-0.520**	-0.539**	-0.612**	-0.473**	-0.535**	-0.571**
	(0.021)	(0.046)	(0.028)	(0.022)	(0.047)	(0.029)	(0.021)	(0.046)	(0.028)
*lskill*_*t*−1_	0.006**	0.005**	0.007**	0.006**	0.004**	0.007**	0.006**	0.005**	0.007**
	(0.001)	(0.001)	(0.001)	(0.001)	(0.001)	(0.001)	(0.001)	(0.001)	(0.001)
*exp*_*t*−1_	-0.028**	-0.022	-0.024*	-0.030**	-0.023	-0.024*	-0.030**	-0.024	-0.027**
	(0.008)	(0.015)	(0.010)	(0.008)	(0.015)	(0.010)	(0.008)	(0.015)	(0.010)
*inn*_*t*−1_	0.048**	0.053**	0.047**	0.049**	0.049**	0.047**	0.048**	0.052**	0.047**
	(0.008)	(0.016)	(0.010)	(0.008)	(0.016)	(0.010)	(0.008)	(0.016)	(0.010)
*lihh*_*t*−1_	0.015**	0.008**	0.014**	0.014**	0.007**	0.013**	0.014**	0.007**	0.014**
	(0.001)	(0.001)	(0.001)	(0.001)	(0.001)	(0.001)	(0.001)	(0.001)	(0.001)
*lmarkup*_*t*−1_	-0.002	0.015	-0.026*	-0.019*	0.009	-0.039**	0.025**	0.054**	0.009
	(0.011)	(0.024)	(0.015)	(0.011)	(0.024)	(0.015)	(0.007)	(0.011)	(0.009)
*lage*_*t*−1_	0.024**	0.015**	0.021**	0.022**	0.015**	0.018**	0.024**	0.016**	0.021**
	(0.003)	(0.005)	(0.004)	(0.003)	(0.005)	(0.004)	(0.003)	(0.005)	(0.004)
*pre_lw*2013	0.155**	0.279**	0.213**	0.182**	0.275**	0.233**	0.151**	0.274**	0.208**
	(0.008)	(0.017)	(0.010)	(0.009)	(0.019)	(0.010)	(0.008)	(0.017)	(0.010)
*loc*_*t*−1_	0.040**	0.060**	0.036**	0.042**	0.059**	0.038**	0.042**	0.061**	0.039**
	(0.006)	(0.016)	(0.009)	(0.007)	(0.016)	(0.009)	(0.006)	(0.016)	(0.009)
*ind/year*	*Yes*	*Yes*	*Yes*	*Yes*	*Yes*	*Yes*	*Yes*	*Yes*	*Yes*
Constant	2.330**	2.138**	2.734**	2.398**	2.141**	2.838**	2.274**	2.086**	2.663**
	(0.105)	(0.220)	(0.131)	(0.104)	(0.198)	(0.136)	(0.107)	(0.225)	(0.135)
Observations	44,272	13,958	30,314	43,187	13,658	29,529	44,299	13,968	30,331
P-value (Wald)	0.000	.0.000	0.000	0.000	.0.000	0.000	0.000	0.000	0.000
Rho	0.087	0.419	0.174	0.131	0.436	0.219	0.085	0.419	0.172

Source: Authors’ own elaboration.

Robust standard error reported in parenthesis.

***, ** and * indicate significance at the 1, 5 and 10%, respectively.

The results are notable. Firstly, all productivity measures positively and statistically significantly impact firm wages. Secondly, the TFP measure without gender differentiation (first three columns) yields lower figures than the gender-specific TFP scenario (columns 4, 5, and 6). Thirdly, as we expected, in this latter scenario, the impact on male-dominated firms is more significant than on female-dominated firms. All else being equal, a 1% increase in TFPwm raises wages by 6.2%, whereas for female firms, the increase is only 5.7%.

The first result eventually supports the neoclassical hypothesis that wages are related to productivity. Nevertheless, in the case of APL (columns 7, 8, and 9), the figures are noticeably lower than those in the first two scenarios. This suggests that using APL to measure a firm’s productivity may be misleading or biased, as it captures short-run employee performance rather than the productivity of the firms as a whole, potentially underestimating the actual impact.

Other covariates reveal additional interesting results. There is evidence of positive wage *persistence* across all productivity measures, indicating that firms that paid higher (or lower) wages in the past are likely to continue doing so in the future. However, regardless of the productivity measure, the impact is consistently more substantial for male firms than for female firms. Furthermore, the positive and statistically significant estimates of the pre-sample mean of wages $\;\left(\mathrm{pr}e_{\mathrm{lw}2013}\right)\;$ , which captures the long-run impact of individual heterogeneity, further support the importance of wages *persistence.* These findings align with the study by Hansen and McNichols (2020), which suggests that employers’ prior knowledge of employees’ salaries perpetuates historical gender-based wage discrimination.

Firm size reveals that regardless of productivity proxy, SMEs show a negative impact on salaries than large firms, and in addition, female firms SMEs reduce the average wages less than male firms SMEs (-0.539 and -0.612 in the TFPwm case, respectively). These results are consistent with Messina (2019), whose findings reveal that large firms are more profitable and, therefore, pay higher wages, and this is not solely because they attract more skilled workers. Employees with identical qualifications earn better salaries when they work for these firms. As well, Blau and Kahn (2000) highlighted that wage disparities between male- and female-dominated firms could be linked to differing management styles, workplace policies, or labor force composition, and some studies suggest that female leadership may be associated with more equitable pay practices.

Moreover, as we expected, the employees’ skills also positively and significantly affect salaries, as predicted by Human Capital theory. Regardless of TFP measurement, the evidence shows female firms have less impact on salaries than male firms, although when the employees have master's degrees. This is a clear signal of gender discrimination corroborated extensively by many authors, such as Rivera-Lozada et al., (2024) for Colombia, in the context of Kitakawa-Oaxaca-Blinder wage discrimination. In addition, in a typical emerging economy, the magnitude of the parameter (elasticity) is less than one. This suggests low education levels among employees and/or a limited number of hires with master’s degrees or that the manufacturing process does not require a highly specialized workforce.

Internationalization strategies indicate that, for exports of final goods, the results for female firms are inconclusive, as the parameter is not significant. Conversely, for process and/or product innovation activities, the findings support the idea of a positive relationship with wages, with the impact being more significant in female firms than male firms.

According to Gómez Sánchez, (2020), this result may be because in Colombia, small firms tend to be more focused on innovation activities than export activities, whereas the opposite trend is observed in large firms. Furthermore, Dezsö and Ross (2012) argue that gender diversity in leadership can enhance team performance, especially in innovation-driven contexts, as diverse perspectives foster solutions that are more creative and improved decision-making.

For female firms, higher concentration levels tend to boost wages, whilst the evidence related to mark-ups is inconclusive, especially in the TFPwm case. When firms hold significant market power, they may share some of their higher profits with employees through increased salaries, potentially to retain talent or improve productivity. Firms’ age positively and significantly affects wages in all scenarios considered. This evidence supports the findings of ILO (2015), where young small businesses are the ones that contribute the most to job creation. Nevertheless, female firms always display a lower impact than firms with a high male proportion.

Lastly, when firms are geographically located in the Capital District of Bogota ( *loc*), which is the most important economic activity zone in Colombia, female firms consistently have a more significant impact than male firms. Rodríguez-Pose and Crescenzi (2008) emphasize the role of regional dynamics, noting that companies located in economically buoyant areas benefit from knowledge diffusion and network effects, which can amplify the influence of various leadership structures, including those led by women.

## Discussion

This document aims to investigate gender wage differentials by exploring the incidence of a manufacturing firm’s productivity on salaries, accounting for gender, in an emerging economy such as Colombia.

The linkage between salaries and a firm's productivity is particularly significant in the Colombian manufacturing industry. Our findings confirm a strong connection between these variables, aligning with the postulates of neoclassical economic theory. However, average labor productivity is inadequate for capturing a firm’s productivity. This metric overlooks crucial complementarities with capital, intermediate inputs, and technology by focusing exclusively on the labor factor. Therefore, Total Factor Productivity (TFP) emerges as a more comprehensive and accurate measure of a firm’s productivity.

Nonetheless, analyzing TFP without disaggregating by gender can obscure critical differences in firms’ productivity. This approach implicitly assumes that all workers contribute homogeneously to production, disregarding structural disparities such as gender gaps in wages, access to training, and working conditions. These omissions can lead to biased interpretations of a firm’s proper productive performance. As previous empirical literature in the industrial organization has largely overlooked the role of gender in TFP measurement, this oversight could result in flawed connections between TFP and variables such as exports, imports, innovation, or ICT, influencing the testing of important hypotheses such as learning by or self-selection.

The wage gaps align with claims made by Goldin (2014) and, more recently, by the World Bank (2024), highlighting women's significant efforts to improve their participation in the labor market. These efforts aim to improve participation rates, productivity, and wage levels. All else being equal, productivity increases in Colombian male firms are associated with higher wage increases than female firms (6.2% > 5.7%). These results are consistent with those of Santos (2017), who claims that wage disparities outweigh productivity differences, contradicting the promise of efficiency theory and evidencing another discriminatory phenomenon in the labor market. This discrimination not only affects women individually through wage disparities but also extends to female-led firms as entities.

However, women often fail to achieve wage increases proportional to their productivity gains. The fact that the labor market allocates higher wages in line with productivity increases predominantly to male firms reveals a discriminatory situation against female firms. Our results, therefore, suggest a possible wage discrimination. Specifically, descriptive statistics reveal that female firms in the Colombian manufacturing industry exhibit higher average productivity than male firms, although average wages show the opposite trend. These findings partially coincide with those of Gomez Sanchez, et al. (2025), who indicate that a higher proportion of female workers leads to higher firm productivity. Furthermore, female firms show higher productivity levels than male firms, and the elasticity of labor output of female employees is higher than that of male employees, indicating that women contribute more to firm output than men.

Finally, the fact that wages in female firms are lower than in male firms, despite their higher productivity, may indicate problems of labor exploitation as well. The results on female firms in Colombia call for further exploration of productivity differences between female and male firms. In particular, it is worth considering whether the productivity gains of female firms are due to the lower wages paid to women. As Santos (2017) postulates, this claim may not be far-fetched given the historical context of the Colombian textile industry, where female firms traditionally paid lower wages to women. Our results indicate that this situation persists over time, suggesting that the productivity of female firms continues to depend on female wage exploitation.

In conclusion, we can point out that gender wage discrimination goes beyond individual differences between men and women to extend to female and male firms in Colombia. This situation could reveal labor exploitation that would be conditioning the productivity of female firms and has been perpetuated since the dawn of industrialization in Colombia. These findings highlight the need for public policies that address gender pay disparities by recognizing and rewarding productivity regardless of gender. These could include gender-sensitive cost accounting systems, incentives for inclusive hiring practices, and targeted efforts to break occupational segregation. Otherwise, persistent wage gaps will continue undermining gender equity in the labor market.

## Ethical considerations

Ethical approval and consent were not required.

## Reporting guidelines

Reporting guidelines were not required. This study is not related to clinical topics.

## Data Availability

Zenodo: EAM-EDIT 3. https://doi.org/10.5281/zenodo.14867593 ( Gómez Sánchez *et al*., 2025). The project contains the following underlying data: EAM-EDIT 3.xlsx. (A merge of two databases: Annual Manufacturing Survey (EAM) and Technological Development and Innovation Survey (EDIT)). Data are available under the terms of the Creative Commons Attribution 4.0 International license (CC-BY 4.0).

## Footnotes

1 Data authored by Gómez Sánchez, A. M., Ramirez, Z., & Rivera Lozada, I. C: EAM-EDIT 3. [Dataset]. *Zenodo.* 2025. https://doi.org/10.5281/zenodo.14867593
