# Productivity, Real Wages, and Gender. A study in Colombian Manufacturing

**DOI:** 10.12688/f1000research.161343.3

**Published:** 2026-09-01

**Authors:** Andrés Mauricio Gómez-Sánchez, Zoraida Ramírez-Gutiérrez, Isabel Cristina Rivera-Lozada

**Affiliations:** 1 Department of Economics, Universidad del Cauca, Popayan, Cauca, Colombia; 2 Department of Accounting, Universidad del Cauca, Popayan, Cauca, Colombia; 3 Department of Economics, Universidad del Cauca, Popayan, Cauca, Colombia

**Keywords:** Wages, Gender, Firms productivity, Manufacturing, Emerging Economy

## Abstract

**Background:**

Orthodox microeconomic theory establishes a positive link between employee wages and productivity in competitive markets. Nevertheless, this perspective often overlooks gender and treats the workforce as a homogeneous category labelled labor, potentially obscuring gender-based inequalities in wage determination. This article addresses the gender wage gap by analyzing, for the first time, at least in an emerging economy such as Colombia, the impact of manufacturing firm’s productivity on wages explicitly including gender.

**Method:**

First, we use the GMM two-equation system proposed by Wooldridge (2009) to obtain consistent and unbiased estimates of output elasticities and TFP, respectively. Secondly, we explore the wages-productivity linkage by gender, implementing a dynamic random effect generalized least squares model (GLS) with panel data to address endogeneity issues.

**Results:**

Our main findings reveal, among others, that firms with a higher proportion of female workers (female-intensive firms) generally have higher productivity than those with a higher proportion of male workers (male-intensive firms).

**Conclusions:**

The effect of female-intensive firm’s productivity on wages is lower than that of male-intensive firm’s productivity, consistent with gender-based wage disparities in the Colombian manufacturing industry.

## Introduction

The economic literature, particularly within the industrial organization, suggests that firm wages depend on several factors, including firm size ( Carlsson, et al. 2016), employee education and skills ( Mincer, 1974; Schultz, 1961), economic subsector ( Suhányi, et al. 2023), geographic location ( Méjean & Patureau, 2010), and the presence or absence of unions ( Card, et al. 2017). Among these determinants, productivity is considered one of the most critical factor influencing wages ( Feldstein, 2008).

Based on the neoclassical microeconomic theory, increases in labor productivity positively affect real wage growth in the long-run ( Mankiw, 2015). In line with this, industrial organization literature acknowledges that firm productivity significantly influences employees’ wages. Although empirical evidence supporting this hypothesis varies in strength depending on the country and industries analyzed, productivity is widely recognized as a key determinant of wages, whether at the individual or firm level ( Meager & Speckesser, 2011).

Despite this, previous literature has left two issues aside. First, the method used to measure productivity and second, the role of gender in these topics. Regarding productivity measurement, several studies, particularly in macroeconomics, have used the average product of labor as a proxy. This approach is limited as it measures labor productivity rather than firm productivity and only applies in the short-run ( Sargent & Rodriguez, 2000). While other studies, particularly in microeconomics, have correctly used total factor productivity (TFP) at the firm level, many studies generate flawed estimates due to the use of statistical methods that generate biased and inconsistent parameters, leading to inaccurate conclusions and recommendations ( Gómez Sánchez, 2020).

The second issue concerns the exclusion of gender from productivity analysis. Microeconomic and industrial organization theory frequently subsumes workers under the generic label of “labor,” a simplification that introduces biases. Factors such as gender-based occupational segregation, unequal access to training, and asymmetric promotion opportunities can distort productivity measurement when labor is treated as a homogeneous input. This limitation is particularly relevant in emerging economies such as Colombia, where industries like Beverages, Garments, Textiles, Pharmaceuticals, and Chemicals employ a large share of women. Recognizing these differences is essential, since productivity may respond differently to female and male participation, and such distinctions should be reflected in wage determination.

In this order of ideas, this paper aims to assess the relationship between wages and productivity in firms with a high proportion of female employees, hereafter referred to as female-intensive firms, compared to firms with a higher proportion of male employees, referred to as male-intensive firms hereafter ( Tsou & Yang, 2019; Gomez Sanchez, et al., 2025). Following Tsou & Yang (2019) and Pfeifer & Wagner (2014), this classification reflects the gender composition of the workforce, not ownership or management structure. To assess this relationship, we first estimate unbiased total factor productivity using the two-stage method proposed by Wooldridge (2009), and for the first time we disaggregate the workforce by gender to obtain a gender-specific productivity estimate rather than an aggregate measure of TFP.

In this regard, we implement the methodology in two steps. First, we use the two-equation system proposed by Wooldridge (2009) to obtain consistent estimates of input elasticities and unbiased TFP estimates. This system is jointly estimated under the Generalised Method of Moments (GMM) framework. As a novelty, we separate employees by gender to estimate TFP. Second, we implement a dynamic random effect generalized least squares model (GLS) with panel data to address endogeneity. Moreover, we also include industrial organization covariates, such as exports or innovations, along with wage persistence; that is, firms with higher (or lower) wages in the past tend to continue those wage levels in the present. In addition, the model also deals with potential initial condition problems ( Blundell & Bond, 1998).

The data come from the Annual Manufacturing Survey (EAM) and the Technological Development and Innovation Survey (EDIT), both published by the Statistics Department of Colombia (DANE) for the period 2013–2020. After merging eight waves of EAM and EDIT, we obtain an unbalanced panel of 59,355 observations.

Our main findings are summarized as follows: i) Female-intensive firms exhibit higher TFP than male-intensive firms. ii) The productivity-wage elasticity in female-intensive firms (0.057%) is lower than in male-intensive firms (0.062%), a pattern consistent with gender-based wage disparities in the Colombian manufacturing industry. iii) The output elasticity of labor in female-intensive firms exceeds that of male-intensive firms, indicating that women contribute proportionally more to firm output than men. iv) TFP displays lower input elasticities than gender-specific TFP estimates, suggesting that aggregate TFP underestimates firm productivity when gender is considered.

The remainder of this paper is organized as follows. Section 2 examines theoretical and empirical literature. Section 3 describes the data. Section 4 displays TFP and stochastic model estimates. Section 5 discusses the results, and Section 6 offers conclusions.

## Methods

### Literature review

To introduce the theoretical foundations linking productivity to wages, several complementary frameworks explain how compensation levels relate to worker and firm performance. Human capital theory argues that individual productivity, and consequently wages, derives from investments in education, training, and work experience ( Schultz, 1961; Mincer, 1974). These investments increase skills and capabilities, producing enduring benefits not only for workers but also for firms through higher efficiency and improved task performance.

Efficiency wage theory, as formulated by Shapiro and Stiglitz (1984) and expanded by Akerlof and Yellen (1986), emphasizes the strategic role of compensation. Firms may deliberately set wages above the market-clearing level to discourage shirking, reduce turnover, and stimulate motivation, thus highlighting the endogenous relationship between remuneration and output. Together, these theories provide a basis for understanding why wages may reflect both the productive potential of workers and managerial choices aimed at improving firm performance. Importantly, this theoretical link also implies a bidirectional relationship: higher wages may themselves increase productivity through motivational channels, a possibility we address explicitly in our empirical strategy (see Empirical Model section).

Building on this foundation, insights from firm heterogeneity and industrial organization literature broaden the discussion by situating productivity within a wider set of market and managerial factors. Syverson (2011) notes that differences in technology adoption, market structure, managerial quality, and access to inputs generate significant productivity gaps among firms operating in similar environments. These findings suggest that productivity does not stem solely from individual characteristics but also from firm-level strategies and competitive pressures that shape the efficiency of resource allocation. As a result, wage determination is influenced not only by the capabilities of workers but also by the organizational and structural conditions under which firms operate.

Incorporating gender economics and labor market segmentation further enriches these models by revealing how institutional and structural biases shape productivity outcomes. Occupational segregation, unequal access to training, and limited promotion prospects systematically distort productivity measures when labor is treated as a homogeneous input ( Blau & Kahn, 2000; Seguino, 2000; England and Folbre, 2002; Kabeer, 2016). A growing body of feminist economics shows that women are frequently concentrated in lower-paid sectors or occupations, creating a persistent gap between their actual contribution to output and their remuneration ( Becker, 1971; Goldin, 2014). Moreover, recent evidence suggests that even when female-intensive firms achieve high productivity, the transmission of that productivity into wages tends to be weaker, pointing to the influence of structural factors ( Rivera-Lozada et al., 2024).

The empirical evidence on gender and productivity presents mixed findings. Tsou and Yang (2019) show that Chinese firms with higher proportions of female workers often display lower productivity than male-intensive firms. Nonetheless, when women possess higher educational attainment, they significantly enhance firm productivity, particularly in small private and foreign enterprises. Pfeifer and Wagner (2014) similarly report contrasting outcomes across estimation methods: under OLS, female -intensive firms appear less productive, yet a GMM framework reverses these findings. Such inconsistencies underscore the importance of methodology and the operational definition of ‘female-intensive’ firms.

Most existing studies focus on the gender of CEOs or owners, whereas our work, constrained by the structure of the EAM survey, classifies firms as female-intensive or male-intensive according to the proportion of women or men in their workforce. Following Tsou and Yang (2019) and Pfeifer and Wagner (2014), we adopt the term female-intensive firms to describe establishments where women represent 50% or more of the total workforce, and male-intensive firms for those where men predominate. This classification reflects labor force composition, not ownership or management structure, a distinction that, as we argue, captures broader organizational practices and sectoral norms that may be invisible in leadership-based classifications.

These dynamics are particularly relevant in emerging economies such as Colombia, where industries including garments, textiles, and pharmaceuticals employ a large share of women ( Ñopo and Gallardo, 2009). Recent Colombian data reinforce this paradox: women-led firms identified in the Unified Business and Social Registry (RUES) represent 59% of the 1.2 million registered enterprises, and according to the Emicron Survey (2022) and WCP (2024), these firms show slightly higher productivity (34.3% compared with 33.25% for male-led firms) and greater efficiency improvements (25.8% versus 17.9%). Nevertheless, wage differentials continue to exceed productivity gaps, suggesting that structural factors, rather than productivity differences alone, account for the persistent disparity.

Lastly, merging these perspectives reveals that the decoupling between wages and productivity, well documented in advanced economies since the 1970s and linked to globalization, declining bargaining power, and labor market segmentation ( Feldstein, 2008; Meager & Speckesser, 2011), acquires a distinctive gendered dimension in emerging economies. In Colombia, this divergence appears amplified for female-intensive firms, suggesting that gender-based wage disparities are embedded in the very mechanisms connecting productivity and remuneration. Understanding these linkages is therefore essential for designing policies that promote fair compensation, enhance firm efficiency, and reduce structural gender inequality.

### TFP estimates by gender

TFP disaggregated by gender follows the two-equation system proposed by Wooldridge (2009). This framework facilitates GMM estimation, improving the efficiency of standard error estimation and eliminating the need for bootstrapping. Specifically, we assume the production process follows a linearized Cobb-Douglas production function:

$$y_{\mathrm{it}}=\beta_{0}+\beta_{\mathrm{lw}}l_{\mathrm{it}}^{w}+\beta_{\mathrm{lm}}l_{\mathrm{it}}^{m}+\beta_{k}k_{\mathrm{it}}+\beta_{m}m_{\mathrm{it}}+\beta_{e}{\mathrm{ene}}_{\mathrm{it}}+\omega_{\mathrm{it}}+\varepsilon_{\mathrm{it}}$$ (1)

where $y_{\mathrm{it}}$ denotes firm *i*'s production in period *t*; $l_{\mathrm{it}}^{w}\;$ is female labor; $l_{\mathrm{it}}^{m}$ is male labour; $k_{\mathrm{it}}\;$ is capital stock; $m_{\mathrm{it}}\;$ is materials consumption; and ${\mathrm{ene}}_{\mathrm{it}}\;$ is electric energy consumption. $\omega_{\mathrm{it}}$ is firm productivity, unobservable to the econometrician but predictable by the firm, and $\varepsilon_{\mathrm{it}}$ is an idiosyncratic error term.

Following Olley and Pakes (1992) and Levinsohn and Petrin (2003), capital stock is a state variable determined in the previous period, so current productivity shocks do not affect capital. Labor by gender and energy consumption are treated as freely variable inputs chosen in the current period. However, Ackerberg, et al. (2015) demonstrate that these input choices are correlated with *ω_it*, rendering OLS, fixed effects (FE), and instrumental variable (IV) estimators biased and inconsistent.

To address this, we use materials demand as a proxy for unobserved productivity ( Levinsohn and Petrin, 2003). Inverting this demand function yields an expression of productivity in terms of observables, which is substituted back into Equation (1) and proxied by third-degree polynomials. Introducing the law of motion of productivity as a Markov process ( Wooldridge, 2009), the two-equation system estimated jointly under GMM is:

$$y_{\mathrm{it}}=\beta_{0}+\beta_{\mathrm{lw}}l_{\mathrm{it}}^{w}+\beta_{\mathrm{lm}}l_{\mathrm{it}}^{m}+\beta_{k}k_{\mathrm{it}}+\beta_{m}m_{\mathrm{it}}+\beta_{e}{\mathrm{ene}}_{\mathrm{it}}+h_{t}\left(k_{\mathrm{it}},\omega_{\mathrm{it}},{\mathrm{ene}}_{\mathrm{it}}\right)+\varepsilon_{\mathrm{it}}$$ (2)

$$y_{\mathrm{it}}=\beta_{0}+\beta_{\mathrm{lw}}l_{\mathrm{it}}^{w}+\beta_{\mathrm{lm}}l_{\mathrm{it}}^{m}+\beta_{k}k_{\mathrm{it}}+\beta_{m}m_{\mathrm{it}}+\beta_{e}{\mathrm{ene}}_{\mathrm{it}}+h\left(k_{\mathrm{it}-1},m_{\mathrm{it}-1},{\mathrm{ene}}_{\mathrm{it}-1}\right)+v_{\mathrm{it}}$$ (3)

where $v_{\mathrm{it}}$ is a composite error term ( $\xi_{\mathrm{it}}+\varepsilon_{\mathrm{it}}$ ). Once the system is estimated, TFP is recovered as the residual:

$${\hat{\omega }}_{\mathrm{it}}=y_{\mathrm{it}}-\left({\hat{\beta }}_{0}+{\hat{\beta }}_{\mathrm{lw}}l_{\mathrm{it}}^{w}+{\hat{\beta }}_{\mathrm{lm}}l_{\mathrm{it}}^{m}+{\hat{\beta }}_{k}k_{\mathrm{it}}+{\hat{\beta }}_{m}m_{\mathrm{it}}+{\hat{\beta }}_{e}{\mathrm{ene}}_{\mathrm{it}}\right)\;$$ (4)

Our key methodological innovation consists in disaggregating the labor factor by gender, estimating separate output elasticities for female labor ( $\beta_{\mathrm{lw}}$ ) and male labour ( $\beta_{\mathrm{lm}}$ ). To our knowledge, this extension of Wooldridge’s (2009) approach to gender-specific TFP has not been implemented in prior studies. This allows us to assess whether women contribute differently to firm output and whether any differential is reflected in wages.

Regarding the limitations of TFP as a measure, we acknowledge the critiques raised by Shaikh (1974), Felipe and McCombie (2020), and related literature. These authors argue that TFP as a residual measure can capture factors beyond technological progress, including income distribution shifts and market power changes. Nonetheless, these critiques operate primarily at the macroeconomic level. Our analysis focuses on firm-level productive efficiency, where TFP is the standard performance measure ( Syverson, 2011). At the microeconomic level, modern production function estimators, including Olley and Pakes (1992), Levinsohn and Petrin (2003), and particularly the two-equation system of Wooldridge (2009), were developed precisely to address endogeneity between inputs and unobserved productivity shocks, yielding more robust and consistent TFP estimates than earlier approaches.

### Data analysis ^1^

The data come from the Annual Manufacturing Survey (EAM) and the Technological Development and Innovation Survey (EDIT), both published by Colombia’s National Statistics Department (DANE) for the period 2013–2020. After merging eight waves of EAM and EDIT, we obtain an unbalanced panel of 59,355 observations. The panel includes 9,715 firms (T = 7 periods on average), covering 23 manufacturing sub-sectors classified according to ISIC Revision 2.

All monetary variables as output, materials, capital, and wages, are deflated to constant 2015 prices using the Industrial Producer Price Index (IPP) for output and material inputs, and the Gross Fixed Capital Formation deflator for capital. Wages are deflated using the Consumer Price Index (CPI, base 2014).

The key variable of interest is the gender composition of the firm’s workforce. Following Tsou and Yang (2019) and Pfeifer and Wagner (2014), and consistent with the classification framework proposed in our methodological note, we define the *female intensity index* ( *fem_index*) as the proportion of women in total employment. We classify a firm as *female-intensive* if *fem_index* ≥ 0.50, and as *male-intensive* otherwise. Robustness checks with alternative thresholds (54.5% and 60%) are reported in the Appendix.

Table 1 presents the estimates of labor product elasticities using three different methods: Ordinary Least Squares (OLS), Generalized Least Squares (GLS), and Wooldridge’s (2009) two-step method. These estimates are analyzed under two scenarios: one considering the firm's total labor force (Labor) and the other distinguishing employees by gender (women and men’s labor).

**Table 1.** Estimates of labor output elasticities. Total labor and labor by gender.

	OLS	GLS	Wooldridge	OLS	GLS	Wooldridge
Labor	0.298***	0.252***	0.286***			
	(0.002)	(0.003)	(0.002)			
Women labor				0.141***	0.100***	0.136***
				(0.001)	(0.002)	(0.001)
Men labor				0.136***	0.137***	0.132***
				(0.002)	(0.003)	(0.002)
Capital	0.042***	0.023***	0.089***	0.045***	0.022***	0.093***
	(0.001)	(0.002)	(0.006)	(0.001)	(0.002)	(0.006)
Materials	0.711***	0.701***	0.684***	0.715***	0.705***	0.690***
	(0.001)	(0.002)	(0.003)	(0.002)	(0.002)	(0.003)
Energy	0.008***	0.056***	0.039***	0.016***	0.061***	0.045***
	(0.001)	(0.002)	(0.003)	(0.001)	(0.002)	(0.003)
Constant	3.096***	3.108***		3.222***	3.257***	
	(0.014)	(0.024)		(0.017	(0.026	
P-value Wald		0.000	0.000		0.000	0.000
P-value F	0.000			0.000		
Observations	67,781	67,781	55,624	65,879	65,879	53,799

Source: Author’s elaboration.

Robust standard error reported in parentheses.

***, ** and * indicate significance at 1, 5 and 10%, respectively.

As expected, the results generally reveal that all methods yield positive and statistically significant elasticities. Nevertheless, without distinguishing by gender, the product elasticity under Wooldridge’s (2009) method is 0.286. In contrast, the OLS and GLS methods tend to overestimate this figure, likely due to biases from unaddressed endogeneity issues in the Cobb-Douglas production function. When gender is considered, Wooldridge’s (2009) method shows that the product elasticity of women’s labor is 0.136, while that of men’s labor is 0.132. The remaining elasticities corresponding to capital (0.093), materials (0.690), and energy (0.045) fall within the ranges reported by empirical studies on the industrial organization in Colombia ( Gómez-Sánchez et al., 2022; Llopis et al., 2022; Sanchis Llopis et al., 2024).

The significance of our findings extends beyond the absolute size of the elasticity gap. The results’ econometric significance stems from their statistical significance and consistency across different model specifications. While the use of logarithmic transformations and monetary variables in U.S. dollars may compress the apparent magnitude, these differences translate into substantial economic effects when converted to Colombian pesos. Furthermore, our findings reveal that the higher output elasticity of female labor is a general characteristic of the Colombian manufacturing industry. This is due to that only one of the twenty-three sectors deviates from the overall pattern.

The differential in labor product elasticities between men and women is noteworthy: the elasticity of female labor is higher, implying that women’s average contribution to the firms’ output surpasses that of men. However, it is possible that women’s wages do not correspond to this greater contribution to production. In this regard, we present additional descriptive analyses to explore this idea.

Figure 1 shows that male-intensive firms have higher average log-wages (13.52) than female-intensive firms (13.09). Besides, in terms of average product of labor (APL), male-intensive firms exhibit higher productivity (11.598) than female-intensive firms (11.127), seemingly aligning with the prediction of neoclassical theory.

**Figure 1.**
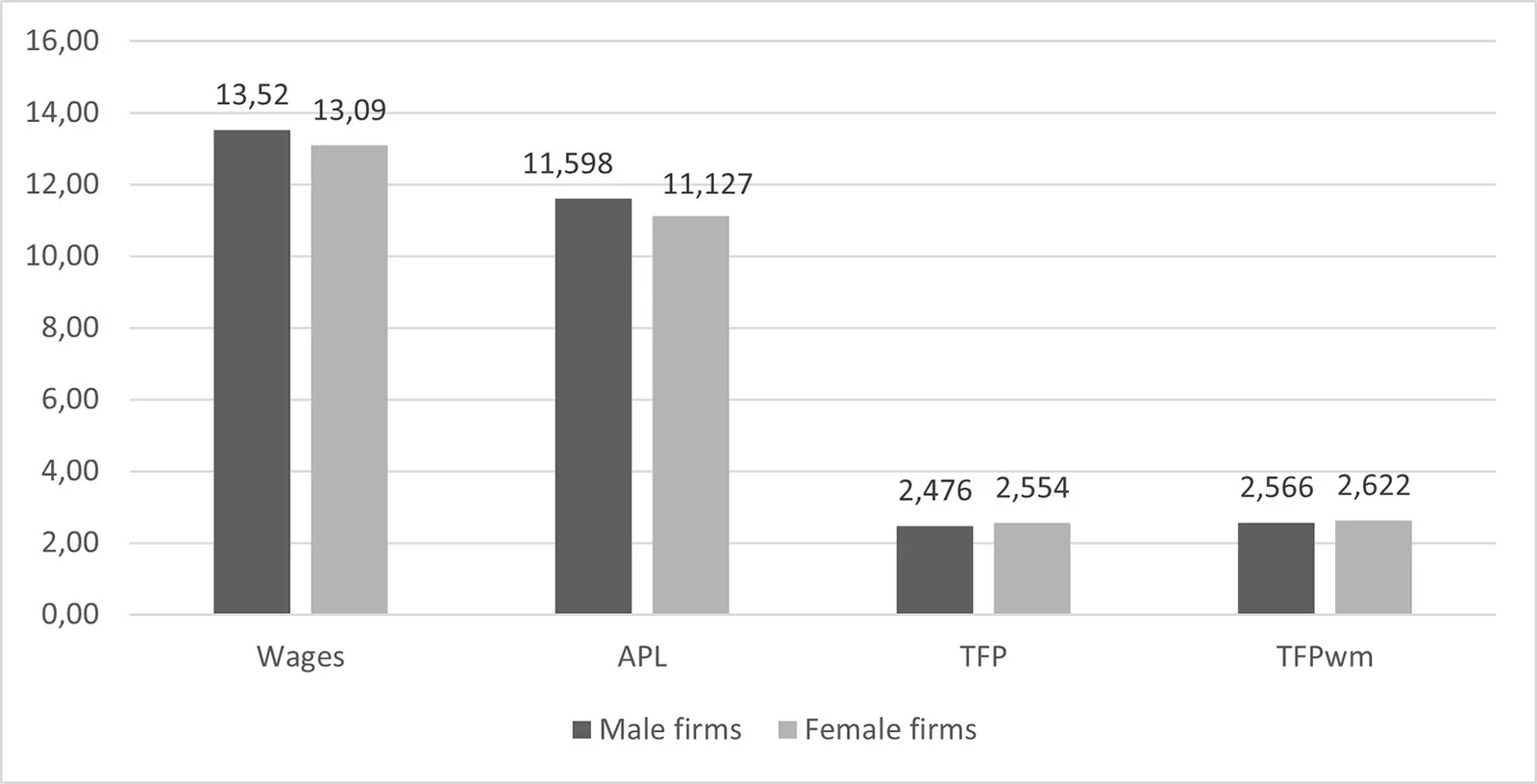
Average logarithmic wages and productivity in female-intensive and male-intensive firms. Source: Authors’ elaboration.

However, more precisely, measures of a firm’s productivity suggest different results. In the TFP scenario, female-intensive firms outperform male-intensive firms, with values of 2.554 and 2.476, respectively. Furthermore, in TFP weighted by gender (TFPwm), female-intensive firms also perform better (2.622) compared to male-intensive firms (2.566). Whilst further analysis is required to establish causality, these findings advise a potential productivity advantage when gender weighting is considered.

Table 2 presents wages and different productivity measures broken down by manufacturing sectors to deepen our analysis. In our preferred scenario (TFPwm), the numbers reveal that in industrial sectors such as Food, Textiles, Leather, Publishing, Coking, chemicals, Electric motors, Vehicles, and Machine Maintenance, female-intensive firms’ productivity displays higher productivity than male-intensive firms. However, wages are lower for female-intensive firms compared to salaries in male -intensive firms. In the remaining sectors, there is a clear correspondence between productivity and wages, that is, the more productive the higher the wages, regardless of firms’ gender orientation.

**Table 2.** Wages and Productivity. Female-intensive and male-intensive firms by sector. Average in Logs.

Industry	Male-intensive Firms				Female-intensive Firms			
ISIC Rev.2	Wages	APL	TFP	TFPwm	Wages	APL	TFP	TFPwm
Food	13.874	12.089	2.437	2.501	12.901	11.179	2.452	2.511
Beverages	14.368	12.127	2.612	2.686	12.856	11.365	2.594	2.640
Textiles	13.874	11.427	2.346	2.416	13.036	11.078	2.493	2.554
Garment	12.809	11.585	2.591	2.637	13.092	10.979	2.625	2.727
Leather	12.739	11.168	2.424	2.495	12.724	10.939	2.487	2.547
Wood	12.838	11.263	2.427	2.554	12.132	10.875	2.398	2.450
Paper	14.310	12.128	2.412	2.486	12.982	11.187	2.418	2.463
Publishing	13.214	11.340	2.470	2.524	12.735	11.152	2.533	2.569
Coking	13.925	13.397	2.758	2.826	12.831	12.910	2.949	2.987
Chemical	13.888	12.273	2.615	2.682	13.665	11.466	2.686	2.750
Pharmaceutical	14.685	12.058	2.838	2.888	14.137	11.508	2.803	2.859
Rubber and Plastic	13.505	11.589	2.377	2.453	13.342	11.212	2.377	2.423
Non-Metallic Mineral Prod	13.823	11.554	2.384	2.522	12.784	11.019	2.404	2.439
Metallurgical Products	13.596	11.844	2.442	2.558	12.298	11.525	2.433	2.465
Metal products	13.178	11.204	2.509	2.630	12.552	10.941	2.472	2.520
Manufacture of Electronics	13.293	11.744	2.643	2.705	12.679	10.941	2.488	2.562
Electric motors	13.900	11.432	2.453	2.545	13.018	11.055	2.509	2.563
Machinery and Equipment	13.305	11.239	2.581	2.699	12.200	11.176	2.563	2.603
Vehicles	13.554	11.460	2.424	2.548	13.479	11.431	2.531	2.607
Ships and Boats	14.052	11.523	2.326	2.434	12.459	11.131	2.378	2.424
Furniture	13.065	11.090	2.456	2.558	12.249	11.078	2.447	2.489
Other manufactures	13.364	11.505	2.541	2.636	13.014	11.075	2.561	2.619
Machine Maintenance	13.596	11.673	2.645	2.772	11.966	11.226	2.792	2.830

Source: Author’s elaboration.

Other productivity measures, such as TFP, are consistent with the observed results for TFPwm. Nonetheless, APL estimates indicate that both productivity and wages are higher in male- intensive firms than in female-intensive firms across all industries except the Garment sector.

### Empirical model and estimates

To examine the relationship between firm productivity and wages by gender composition, we estimate a dynamic generalized least squares (GLS) model with random effects (RE) for panel data. All variables are expressed in natural logarithms except dummy variables. To address simultaneity, that is, the possibility that wages contemporaneously affect productivity through efficiency wage mechanisms and vice versa, all covariates are lagged one period. The model is:

$$lw_{\mathrm{it}}=\varphi^{0}+\alpha^{0}lw_{\mathrm{it}-1}+\alpha^{1}\text{find}x_{\mathrm{it}-1}^{q,r}+\alpha^{2}\text{lpro}d_{\mathrm{it}-1}^{h,j,p}+\gamma Z_{\mathrm{it}-1}+\mathrm{pre}\_\mathrm{lw}2013+\mathrm{lo}c_{j}+\mathrm{yea}r_{t}+\text{in}d_{j}+\varepsilon_{\mathrm{it}\;}$$ (5)

where $lw_{\mathrm{it}}$ is the firm’s average real wage (in logs); $\text{find}x_{\mathrm{it}-1}^{q,r}$ is the *female intensity index* (continuous or dichotomous); $\text{lpro}d_{\mathrm{it}-1}^{h,j,p}$ denotes firm productivity ( *h* = aggregate TFP, *j* = gender-weighted TFP, *p* = APL); and $Z_{\mathrm{it}-1}$ is a vector of firm-level controls. The lagged dependent variable $lw_{\mathrm{it}-1}$ captures wage persistence, that is, firms with higher (or lower) wages in the past continue to display higher (or lower) wages in the present ( Roberts and Tybout, 1997). In our context, this persistence may reflect structural gender-based disparities, as firms that historically paid lower wages to workers in *female-intensive firms* continue to do so. The term pre_lw2013 (pre-sample means of wages, Blundell and Bond, 1998) controls for long-run unobserved firm heterogeneity correlated with wage levels. ^2^

The vector $Z_{\mathrm{it}-1}\;$ includes firms characteristics such as firm size (SMEs dummy, ≤200 employees); employee skills (number of workers with master’s degrees) as a proxy for human capital ( Schultz, 1961; Mincer, 1974); export status and innovation activities (process and product combined) as internationalization and innovation proxies ( De Loecker, 2013; Crepon et al., 1998); the Herfindahl-Hirschman Index (HHI) for market concentration; firm-level mark-up; and firm age. We also control for location *loc*_*j*_ (Bogotá D.C.), macroeconomic shocks (year fixed effects, *year*_*t*_), and sector characteristics (industry fixed effects, *ind*_*j*_).

*Specification Tests and Robustness*

Our estimation strategy was subject to three complementary specification tests regarding the econometric justification for the random-effects specification.

*Serial Correlation Test*

As a first diagnostic, we apply the Wooldridge (2002) test for first-order serial correlation in panel data. Results show strong evidence of autocorrelation in the static (non-dynamic) models for all three productivity specifications, *TFPwm* (p < 0.001), aggregate TFP (p < 0.001), and APL (p <0.001). This finding *validates* our dynamic specification. The strong serial correlation in wages, consistent with wage rigidity documented across labor markets, provides direct empirical justification for including the lagged wage $lw_{\mathrm{it}-1}\;$ as a regressor. Our dynamic model absorbs this autocorrelation, ensuring residuals are serially uncorrelated and estimates are consistent.

*Hausman Test*

Table A1 reports the Hausman specification test comparing fixed-effects (FE) and random-effects (RE) estimators for the preferred *TFPwm* specification. The test formally rejects RE in all three subsamples, full sample, *female-intensive*, and male-intensive all with p < 0.001. Nevertheless, this test result must be interpreted with caution in dynamic panel models.

When the lagged dependent variable $lw_{\mathrm{it}-1}$ is included as a regressor, the within (FE) estimator is also inconsistent, this is the well-known *Nickell (1981)* bias. The bias arises because the within-group transformation creates a negative correlation between $lw_{\mathrm{it}-1}\;$ and the transformed error term, which remains even as N tends to infinity and disappears only as T tends to infinity.

With *T* = 7 periods in our sample, this bias is non-trivial. The evidence is visible in Table A1 because the lagged wage coefficient under FE (0.33–0.38) is lower than under RE (0.59-0.67), a discrepancy of approximately - 0.29 that is Nickell bias rather than a structural difference between estimators. As a result, the Hausman test is comparing two inconsistent estimators, and is therefore uninformative in this context (Wooldridge, 2010).

*Correlated Random Effects (CRE)*

To provide a valid test of the RE orthogonality assumption, we follow Mundlak (1978) and Chamberlain (1980) framework and estimate Correlated Random Effects (CRE) models. This approach is appropriate precisely in dynamic panel settings where the standard Hausman test is invalid (Wooldridge, 2010). The CRE model relaxes the strict RE assumption by modelling the correlation between unobserved firm heterogeneity ( $u_{i}$ ) and time-varying regressors explicitly:

$$u_{i}=\alpha +\lambda {\bar{X}}_{i}+v_{i},\;\text{where}\;v_{i}\;⊥\;X_{\mathrm{it}}$$ (6)

where ${\bar{X}}_{i}\;$ are firm-level time averages of all time-varying lagged regressors ( *productivity, skills, market concentration, mark-up, exports, innovation, age,* and *firm size*) and $v_{i}\;$ is orthogonal to regressors by construction. Including ${\bar{X}}_{i}$ in the RE model yields estimates that are consistent even if $u_{i}$ is correlated with $X_{\mathrm{it}}$ . A joint Wald test on the coefficients of ${\bar{X}}_{i}\;$ provides the specification test, that is, non-rejection supports the original RE specification; rejection indicates correlated heterogeneity but does not invalidate the CRE estimates themselves.

Table A2 in the appendix reports RE and CRE estimates side by side for the preferred *TFPwm* specification. Three findings are noteworthy. First, the joint Wald test strongly rejects the null that all Mundlak terms are zero, that is, chi2(8) = 1,320.30 ( *full sample*), 395.15 ( *female-intensive*), and 977.63 ( *male-intensive*), all p < 0.001. This confirms that unobserved firm heterogeneity is correlated with time-varying regressors, as expected in any sample of heterogeneous manufacturing firms. The primary driver of this rejection is between-firm variation in productivity ( *m_ltfp_wm* = −0.216*** to −0.319***), market concentration ( *m_lihh* = 0.071***−0.079***), and mark-up ( *m_lmarkup* = 0.247***−0.289***). These between-firm differences are large and structurally determined, reflecting persistent sorting of firms into different productivity and wage regimes.

Second, and crucially for our main argument, the key coefficient *ltfp_wm _(t-1)_* in the CRE model reveals an asymmetry by gender composition. For *male-intensive firms*, the within-firm (transitory) effect of TFP on wages becomes non-significant in the CRE model (0.018, *p* > 0.10), indicating that the RE estimate was partly driven by between-firm heterogeneity. For *female-intensive firms*, the within-firm effect remains statistically significant and is in fact larger in the CRE model (0.087*, *p* < 0.10) than in the RE estimate (0.057*). This finding strengthens the paper’s core argument, as the productivity-wage link is not only weaker for *female-intensive* firms in cross-sectional terms but also operates through a distinct dynamic channel within firms over time.

Third, the wage persistence is strong and positive in all specifications, also SMEs pay lower wages than large firms, besides skills, innovation, and geographic location significantly affect wages, lastly the differential between *female-intensive* and *male-intensive firms* in the productivity-wage link is robust.

*Robustness to Alternative Classification Thresholds*

Table A3 of the appendix presents the main coefficient of interest ( $\text{ltfp}\_{\mathrm{wm}}_{t-1}$ ) under alternative thresholds for classifying firms as *female-intensive* (≥50%, ≥54.5%, ≥60%). For *male-intensive firms*, results are virtually identical across all thresholds (0.061–0.062***), demonstrating full robustness. For *female-intensive firms*, the coefficient ranges from 0.057* to 0.047 across thresholds. The slight loss of significance at higher thresholds reflects reduced sample sizes (13,658 to 10,887 to 8,768 observations) rather than structural instability, as the point estimates remain close. This pattern is consistent with a productivity-wage link in *female-intensive* firms that becomes harder to estimate precisely as the sample falls.

The difference in the productivity-wage elasticity between *male-intensive firms* (0.062%) and *female-intensive firms* (0.057%) implies that, for every 1% increase in TFP, male-intensive firms transfer approximately 0.005 percentage points more into wages. Expressed in Colombian pesos, given average TFP and wage levels in the sample, this differential amounts to a meaningful and economically relevant gap, reinforcing the hypothesis of *gender-based wage disparities*.

## Results

Table 3 shows the estimates of the empirical modelling. Columns (1), (2), and (3) display TFP results for the full industry, *female-intensive firms*, and *male-intensive firms*, respectively. Columns (4), (5), and (6) present TFP results classified by workforce gender, whilst the final three columns analyze the average product of labor (APL). Each scenario includes continuous and dichotomous indices for *female-intensive firms* and wage persistence.

**Table 3.** Empirical Estimates.

		*TFP*			*TFPwm*			*APL*	
	*Industry*	*Female firm*	*Male firm*	*Industry*	*Female firm*	*Male firm*	*Industry*	*Female firm*	*Male firm*
*lrw*_*t*−1_	0.701**	0.587**	0.619**	0.668**	0.591**	0.592**	0.701**	0.587**	0.619**
	(0.010)	(0.019)	(0.012)	(0.010)	(0.020)	(0.012)	(0.010)	(0.019)	(0.012)
*ltfp*_*t*−1_	0.038**	0.055*	0.050**						
	(0.015)	(0.031)	(0.019)						
${\mathrm{ltfp}}_{t-1}^{\mathrm{wm}}$				0.059**	0.057*	0.062**			
				(0.015)	(0.031)	(0.019)			
*lapl*_*t*−1_							0.016**	0.021*	0.021**
							(0.005)	(0.010)	(0.006)
*smes*_*t*−1_	-0.476**	-0.535**	-0.574**	-0.520**	-0.539**	-0.612**	-0.473**	-0.535**	-0.571**
	(0.021)	(0.046)	(0.028)	(0.022)	(0.047)	(0.029)	(0.021)	(0.046)	(0.028)
*lskill*_*t*−1_	0.006**	0.005**	0.007**	0.006**	0.004**	0.007**	0.006**	0.005**	0.007**
	(0.001)	(0.001)	(0.001)	(0.001)	(0.001)	(0.001)	(0.001)	(0.001)	(0.001)
*exp*_*t*−1_	-0.028**	-0.022	-0.024*	-0.030**	-0.023	-0.024*	-0.030**	-0.024	-0.027**
	(0.008)	(0.015)	(0.010)	(0.008)	(0.015)	(0.010)	(0.008)	(0.015)	(0.010)
*inn*_*t*−1_	0.048**	0.053**	0.047**	0.049**	0.049**	0.047**	0.048**	0.052**	0.047**
	(0.008)	(0.016)	(0.010)	(0.008)	(0.016)	(0.010)	(0.008)	(0.016)	(0.010)
*lihh*_*t*−1_	0.015**	0.008**	0.014**	0.014**	0.007**	0.013**	0.014**	0.007**	0.014**
	(0.001)	(0.001)	(0.001)	(0.001)	(0.001)	(0.001)	(0.001)	(0.001)	(0.001)
*lmarkup*_*t*−1_	-0.002	0.015	-0.026*	-0.019*	0.009	-0.039**	0.025**	0.054**	0.009
	(0.011)	(0.024)	(0.015)	(0.011)	(0.024)	(0.015)	(0.007)	(0.011)	(0.009)
*lage*_*t*−1_	0.024**	0.015**	0.021**	0.022**	0.015**	0.018**	0.024**	0.016**	0.021**
	(0.003)	(0.005)	(0.004)	(0.003)	(0.005)	(0.004)	(0.003)	(0.005)	(0.004)
*pre_lw*2013	0.155**	0.279**	0.213**	0.182**	0.275**	0.233**	0.151**	0.274**	0.208**
	(0.008)	(0.017)	(0.010)	(0.009)	(0.019)	(0.010)	(0.008)	(0.017)	(0.010)
*loc*_*t*−1_	0.040**	0.060**	0.036**	0.042**	0.059**	0.038**	0.042**	0.061**	0.039**
	(0.006)	(0.016)	(0.009)	(0.007)	(0.016)	(0.009)	(0.006)	(0.016)	(0.009)
*ind/year*	*Yes*	*Yes*	*Yes*	*Yes*	*Yes*	*Yes*	*Yes*	*Yes*	*Yes*
Constant	2.330**	2.138**	2.734**	2.398**	2.141**	2.838**	2.274**	2.086**	2.663**
	(0.105)	(0.220)	(0.131)	(0.104)	(0.198)	(0.136)	(0.107)	(0.225)	(0.135)
Observations	44,272	13,958	30,314	43,187	13,658	29,529	44,299	13,968	30,331
P-value (Wald)	0.000	.0.000	0.000	0.000	.0.000	0.000	0.000	0.000	0.000
Rho	0.087	0.419	0.174	0.131	0.436	0.219	0.085	0.419	0.172

Source: Authors’ own elaboration.

Robust standard error reported in parenthesis.

***, ** and * indicate significance at the 1, 5 and 10%, respectively.

Firstly, the results show that all productivity measures positively and statistically significantly impact firm wages. Secondly, the TFP measure without gender differentiation (first three columns) yields lower elasticities than the gender-specific TFP scenario (columns 4, 5, and 6). Thirdly, as expected, in this latter scenario, the productivity-wage elasticity for *male-intensive firms* is higher than for *female-intensive firms*. All else being equal, a 1% increase in *TFPwm* is associated with a 0.062% increase in real wages for *male-intensive firms*, compared to 0.057% for *female-intensive firms*. The first result supports the neoclassical hypothesis that wages are related to productivity. Nevertheless, in the case of APL (columns 7, 8, and 9), the elasticities are noticeably lower than those in the first two scenarios. This suggests that using APL to measure a firm’s productivity may be misleading or biased, as it captures short-run employee performance rather than the productivity of the firm, potentially underestimating the actual impact.

Other covariates reveal additional interesting results. There is evidence of positive wage persistence across all productivity measures, indicating that firms that paid higher (or lower) wages in the past are likely to continue doing so in the future. However, regardless of the productivity measure, the impact is consistently more substantial for *male-intensive firms* than for *female-intensive firms*. Furthermore, the positive and statistically significant estimates of the pre-sample mean of wages ( *pre_lw2013*), which captures the long-run impact of individual heterogeneity, further support the importance of wage persistence. These findings align with the study by Hansen and McNichols (2020), which suggests that employers’ prior knowledge of employees’ salaries perpetuates historical gender-based wage disparities.

Firm size reveals that, regardless of productivity proxy, SMEs show a negative impact on salaries compared to large firms. In addition, female-intensive firms’ SMEs reduce average wages less than *male-intensive firms'* SMEs (-0.539 and -0.612 in the *TFPwm* case, respectively). These results are consistent with Messina (2019), whose findings reveal that large firms are more profitable and therefore pay higher wages, and this is not solely because they attract more skilled workers. Employees with identical qualifications earn better salaries when they work for these firms. Blau and Kahn (2000) highlighted that wage disparities between *male- and female-intensive firms* could be linked to differing management styles, workplace policies, or labor force composition, and some studies suggest that female leadership may be associated with more equitable pay practices.

Moreover, as expected, employees’ skills also positively and significantly affect salaries, as predicted by Human Capital theory. Regardless of TFP measurement, the evidence shows *female-intensive firms* have less impact on salaries than *male-intensive firms* when employees hold master’s degrees. These patterns are consistent with gender-based wage disparities documented extensively by many authors, such as Rivera-Lozada et al. (2024) for Colombia, in the context of the Kitagawa-Oaxaca-Blinder decomposition. In addition, in a typical emerging economy, the magnitude of the parameter (elasticity) is less than one. This suggests low education levels among employees and/or a limited number of hires with master’s degrees, or that the manufacturing process does not require a highly specialized workforce.

Internationalization strategies indicate that, for exports of final goods, the results for *female-intensive firms* are inconclusive, as the parameter is not significant. Conversely, for process and/or product innovation activities, the findings support the idea of a positive relationship with wages, with the impact being more significant *in female-intensive firms* than *in male-intensive firms*. According to Gómez Sánchez (2020), this result may be because in Colombia, small firms tend to be more focused on innovation activities than export activities, whereas the opposite trend is observed in large firms. Furthermore, Dezsö and Ross (2012) argue that gender diversity in leadership can enhance team performance, especially in innovation-driven contexts, as diverse perspectives foster more creative solutions and improved decision-making.

For *female-intensive firms*, higher concentration levels tend to boost wages, whilst the evidence related to mark-ups is inconclusive, especially in the *TFPwm* case. When firms hold significant market power, they may share some of their higher profits with employees through increased salaries, potentially retaining talent or improving productivity. Firms’ age positively and significantly affect wages in all scenarios considered. This evidence supports the findings of ILO (2015), where young small businesses are the ones that contribute the most to job creation. Nevertheless, *female-intensive firms* always display a lower impact than *male-intensive firms*.

Lastly, when firms are geographically located in the Capital District of Bogotá ( *loc*), which is the most important economic activity zone in Colombia, *female-intensive firms* consistently have a more significant impact than *male-intensive firms*. Rodríguez-Pose and Crescenzi (2008) emphasize the role of regional dynamics, noting that companies located in economically buoyant areas benefit from knowledge diffusion and network effects, which can amplify the influence of various leadership structures, including those led by women.

## Discussion

The relationship between productivity and wages in the Colombian manufacturing industry confirms that, although both variables are linked, the transmission of productivity into wages differs significantly depending on the gender composition of firms. Our findings show that the marginal contribution of female labor to output is greater than that of male labor, which aligns with evidence from other emerging economies ( Tsou and Yang, 2019; Pfeifer and Wagner, 2014). However, this higher contribution is not proportionally reflected in remuneration, supporting the hypothesis that structural discrimination mechanisms persist in labor markets ( Blau and Kahn, 2000; Seguino, 2000).

While the difference in output elasticities (0.004 percentage points) and productivity-wage elasticities (0.062% versus 0.057% per 1% increase in *TFPwm*) appears numerically small, these differentials accumulate over the eight-year panel period and, expressed in Colombian pesos at average wage and productivity levels, translate into economically meaningful wage gaps, particularly in the high-productivity sectors identified in Table 2. We acknowledge that the wages-productivity relationship may also operate in the reverse direction through efficiency wage channels ( Akerlof and Yellen, 1986): higher wages may themselves incentivize effort and raise productivity. Our dynamic specification, with all regressors lagged one period and wage persistence explicitly modelled, addresses this simultaneity by isolating the within-firm effect of past productivity on current wages.

This result has two major implications. First, it challenges the predictions of orthodox microeconomic theory, which assumes a neutral transmission between productivity and wages ( Mankiw, 2015). Second, it highlights the importance of incorporating feminist economics and labor segmentation approaches, which emphasize how occupational segregation, unequal access to training, and career progression barriers distort the productivity-wage relationship ( Kabeer, 2016; England and Folbre, 2002; Ñopo and Gallardo, 2009). These perspectives suggest that productivity cannot be treated as a gender-neutral input, especially in economies where women are concentrated in specific industries.

Importantly, this conclusion is confirmed by the Correlated Random Effects (CRE) robustness analysis (Table A2) as even after fully controlling for firm-level unobserved heterogeneity, the within-firm productivity-wage elasticity for *female-intensive firms* remains statistically significant and is in fact larger in the CRE model (0.087*) than in the baseline RE specification (0.057*). For *male-intensive firms*, the within-firm effect is primarily structural, driven by between-firm differences, which itself reveals an asymmetric transmission mechanism by gender composition.

At the same time, some limitations of this study should be considered. First, due to data constraints, the classification of firms as *female-intensive or male-intensive* relies on the proportion of female workers in the workforce, not on ownership or leadership. While this approach is consistent with the empirical literature ( Tsou and Yang, 2019; Pfeifer and Wagner, 2014) and is explicitly motivated in our methodology, it may complicate direct comparisons with studies that classify firms by CEO or owner gender.

Second, although the econometric strategy (Wooldridge, 2009) addresses endogeneity in the production function, productivity measures remain sensitive to sectoral data availability and potential measurement errors ( Felipe & McCombie, 2020). Third, the analysis is restricted to manufacturing firms, while in service sectors, where female employment is even higher, patterns of gender-based wage disparities may diverge ( World Bank, 2024). Fourth, and crucially, our firm-level panel specification identifies correlations rather than causal mechanisms. Establishing that observed wage gaps constitute discrimination in the legal or precise economic sense requires worker-level data, matched employer-employee datasets, or quasi-experimental identification strategies (e.g., minimum wage reforms or trade liberalization episodes) that allow ruling out alternative explanations such as compensating differentials or unobserved skill sorting. Our findings should therefore be interpreted as evidence consistent with gender-based wage disparities, not as definitive proof of intentional discrimination.

Fifth, the robustness concern regarding the random effects specification has been addressed through a Correlated Random Effects (CRE/Mundlak) analysis reported in Table A2 of the Appendix. The CRE estimates confirm that the main results are robust to controlling for firm-level unobserved heterogeneity, and the Nickell (1981) bias in the Hausman test is explicitly documented.

A sixth limitation concerns the role of labor market informality, which this study does not explicitly account for. Colombia, like most Latin American economies, exhibits persistently high levels of informal employment, approximately 57% of the labor force during the study period (DANE, 2020; La Porta & Shleifer, 2014). Critically, informality is not gender-neutral as women are systematically more exposed to informal employment than men, with female informality rates consistently exceeding male rates across all Colombian regions and sectors (ILO, 2018; Perry et al., 2007).

This gender-differentiated exposure to informality has two direct implications for our findings. First, the EAM survey covers only formal manufacturing establishments with ten or more workers, meaning that informal workers (who are disproportionately female) are excluded from our sample. This may lead to an underestimation of the true extent of gender-based wage disparities in Colombian manufacturing. Second, high economy-wide informality can suppress wages even among formal workers through labor market segmentation, that is, informal employment acts as an outside option that weakens workers’ bargaining power, particularly for women, thereby contributing to a weaker productivity–wage pass-through in *female-intensive firms* independently of firm-level discrimination mechanisms (Maloney, 2004; Perry et al., 2007; Ohnsorge & Yu, 2022). Incorporating informality, through linked employer–employee datasets that span both formal and informal employment, represents a direction for future research on gender wage gaps in Colombian manufacturing.

Notwithstanding these limitations, the findings carry important practical implications. Narrowing the gap between productivity and wages in female-intensive firms is not only a matter of equity but also of efficiency. Ignoring the productive contribution of female labor constrains firm competitiveness and weakens inclusive economic growth.

Policy initiatives could include the adoption of gender-disaggregated cost accounting systems, fiscal incentives for firms with inclusive hiring and promotion practices, and training programs targeting women in high-productivity sectors ( ILO, 2015; Rodríguez-Pose & Crescenzi, 2008). Such measures would contribute to aligning wages with productivity while addressing structural sources of gender inequality.

In other words, the persistence of a productivity-wage gap in *female-intensive firms* demonstrates that gender-based wage disparities are not incidental but structurally embedded in the mechanisms linking productivity and remuneration. Advancing towards policies and business practices that recognize the differential contribution of female labor is crucial not only for achieving gender equality but also for strengthening the efficiency and sustainability of the Colombian productive sector.

## Conclusions

As a conclusion, we can point out that gender-based wage disparities extend beyond individual differences between men and women to encompass *female-intensive* and *male-intensive firms* in Colombia. This situation may reflect structural wage inequality that may constrain the productivity potential of *female-intensive firms* and has persisted since the early stages of industrialization in Colombia. These findings highlight the need for public policies that address gender pay disparities by recognizing and rewarding productivity regardless of gender. These could include gender-sensitive cost accounting systems, incentives for inclusive hiring practices, and targeted efforts to break occupational segregation. Otherwise, persistent wage gaps will continue undermining gender equity in the labor market.

## Ethical considerations

Ethical approval and consent were not required.

## Reporting guidelines

Reporting guidelines were not required. This study is not related to clinical topics.

## Data Availability

Zenodo: EAM-EDIT 3. https://doi.org/10.5281/zenodo.14867593 ( Gómez Sánchez *et al*., 2025). The project contains the following underlying data: EAM-EDIT 3.xlsx. (A merge of two databases: Annual Manufacturing Survey (EAM) and Technological Development and Innovation Survey (EDIT)). Data are available under the terms of the Creative Commons Attribution 4.0 International license (CC-BY 4.0).

## Footnotes

1
Data authored by Gómez Sánchez, A. M., Ramirez, Z., & Rivera Lozada, I. C: EAM-EDIT 3. [Dataset]. *Zenodo.* 2025. https://doi.org/10.5281/zenodo.14867593.

2
Although System GMM ( Blundell & Bond, 1998) is an alternative for dynamic panels, our specification addresses the initial conditions problem, the primary motivation for GMM, through the inclusion of pre-sample means of the dependent variable (pre_lw2013), following the parametric approach proposed by Wooldridge (2005). This strategy controls for the correlation between the lagged wage and the individual fixed effect without the instrument proliferation concerns that arise in System GMM when *T* is moderate ( $\overset{-}{T}$ ≈ 7 periods). The CRE/Mundlak estimates reported in Table A2 of the Appendix further confirm that the main results are robust to alternative treatments of unobserved firm heterogeneity, providing additional validation of our econometric strategy.
