# Supplementary material for: Productivity, Real Wages, and Gender. A study in Colombian Manufacturing
Source: F1000Res. 2026 Sep 1;14:306. Originally published 2025 Mar 17. [Version 3] doi: 10.12688/f1000research.161343.3 (PMC13674784; doi:10.12688/f1000research.161343.3)
Supplement: Peer review history

# Reviewer report: Reviewer response for version 3

**DOI:** 10.5256/f1000research.207118.r516938

**Published:** 2026-09-16

**Authors:** Davide Villani

**Affiliations:** 1 Joint Research Centre, European Commission, Seville, Spain

To me the paper is approved, the authors have addressed successfully the comments raised by the reviewers.

Is the work clearly and accurately presented and does it cite the current literature?

Partly

If applicable, is the statistical analysis and its interpretation appropriate?

Partly

Are all the source data underlying the results available to ensure full reproducibility?

Partly

Is the study design appropriate and is the work technically sound?

No

Are the conclusions drawn adequately supported by the results?

Partly

Are sufficient details of methods and analysis provided to allow replication by others?

No

Reviewer Expertise:

NA

I confirm that I have read this submission and believe that I have an appropriate level of expertise to confirm that it is of an acceptable scientific standard.

# Reviewer report: Reviewer response for version 2

**DOI:** 10.5256/f1000research.197171.r478893

**Published:** 2026-05-14

**Authors:** Sara Caria

**Affiliations:** 1 University of Modena and Reggio Emilia, Reggio Emilia, Italy

The paper analyzes how firm productivity relates to wages and workforce gender composition in Colombian manufacturing firms using panel data from EAM and EDIT (2013–2020). It combines a Wooldridge-type production function with a dynamic random-effects wage model to assess whether productivity gains translate into wages differently across firms with varying gender compositions. The study contributes to the literature on labor, industrial organization, gender, and productivity in emerging economies, and proposes a methodological extension by incorporating gender-disaggregated labor inputs into TFP estimation.

 The topic is important and the paper has potential to make a valuable contribution. However, some revisions could improve the quality of the manuscript.

 In the first place, the paper overlooks completely any reference to informality, which represent a high share of the labor market in Colombia, as in the rest of Latin America. High levels of informality affect not only informal workers but also outcomes in the formal sector through several interconnected channels. Women are far more exposed to informality than men, which should at least be mentioned as a potential part of the explanation. There is a consolidated literature showing that high informality contributes to weaker productivity–wage pass-through, greater labor market segmentation, and lower wage growth even among formal workers.

 Some other suggestions are:

 1.The classification of firms based on the share of female employees is translated into concepts such as female ownership, management, or leadership, in a way that is unclear and sometimes such classifications overlap. A high share of female workers within a firm may simply reflect its presence in sectors that are typically lower-paying or more labor-intensive, rather than any gender-related characteristics of firm control or decision-making. Please revise the terminology, explain the different concepts and use them consistently.

 2. Some coefficient interpretations in the manuscript appear overstated. For instance, coefficients around 0.05–0.06 are interpreted as implying that a 1% increase in productivity leads to a 5–6% increase in wages, which is not consistent with the standard interpretation of log-log models. In addition, while the difference between female and male labor elasticities (0.136 versus 0.132) is statistically significant, its economic magnitude may be not so relevant. The manuscript would benefit from more clearly distinguishing between statistical significance and economic relevance, and from providing a more careful discussion of the practical size of the estimated effects.

 3. Justification for the selection of random effect could be strengthened.

Is the work clearly and accurately presented and does it cite the current literature?

Partly

If applicable, is the statistical analysis and its interpretation appropriate?

Partly

Are all the source data underlying the results available to ensure full reproducibility?

Yes

Is the study design appropriate and is the work technically sound?

Partly

Are the conclusions drawn adequately supported by the results?

Partly

Are sufficient details of methods and analysis provided to allow replication by others?

Yes

Reviewer Expertise:

gender gap, labour market dynamics, collective bargaining

I confirm that I have read this submission and believe that I have an appropriate level of expertise to confirm that it is of an acceptable scientific standard, however I have significant reservations, as outlined above.

# Response

**Published:** 2026-07-16

**Authors:** Zoraida Ramírez-Gutiérrez

**Affiliations:** Cauca, Universidad del Cauca, Popayán, Cauca, Colombia

Dear Reviewer,

 Thank you for your careful reading of our manuscript and for your constructive comments. Your observations are well-founded and have led to meaningful improvements in the paper. Below we provide a detailed response to each of your points, indicating the specific changes made in the revised version.

**Comment 1:** *The paper overlooks completely any reference to informality, which represents a high share of the labor market in Colombia, as in the rest of Latin America. High levels of informality affect not only informal workers but also outcomes in the formal sector through several interconnected channels. Women are far more exposed to informality than men, which should at least be mentioned as a potential part of the explanation. There is a consolidated literature showing that high informality contributes to weaker productivity–wage pass-through, greater labor market segmentation, and lower wage growth even among formal workers.*

**Reply 1:**

 We fully agree with this observation. Informality is a structural feature of the Colombian labor market that our study did not address, and we appreciate the Reviewer bringing this gap to our attention.

 Colombia exhibits one of the highest informality rates in Latin America, with approximately 57% of the total labor force in informal employment during the study period (DANE, 2020; La Porta & Shleifer, 2014). Critically, this burden is not gender-neutral: female informality rates consistently exceed male rates across all Colombian regions and sectors (ILO, 2018; Perry et al., 2007). This has two direct implications for our analysis.

 First, the EAM survey, our primary data source, covers only formal manufacturing establishments with ten or more workers. Informal workers, who are disproportionately female, are therefore excluded from the sample. This structural exclusion may lead to an underestimation of the full extent of gender-based wage disparities in Colombian manufacturing, as the most economically vulnerable female workers do not appear in our data.

 Second, and as the Reviewer correctly highlights, high economy-wide informality can suppress wages even among formal workers through labor market segmentation. Informal employment serves as an outside option that weakens workers' bargaining power, particularly for women, whose concentration in informal sectors reduces their leverage in formal wage negotiations (Maloney, 2004; Perry et al., 2007; Ohnsorge & Yu, 2022). This channel is consistent with our finding of a weaker productivity–wage pass-through in female-intensive firms, and represents a complementary, rather than competing, explanation to the structural gender-based disparities we document.

 In this sense, we have added a sixth limitation paragraph in the Discussion section, explicitly acknowledging the role of informality, its gender dimension, and its implications for the interpretation of our findings. Five new references were added to the bibliography: DANE (2020), ILO (2018), La Porta & Shleifer (2014), Maloney (2004), Ohnsorge & Yu (2022), and Perry et al. (2007).

 The new paragraph reads as follows:

*A sixth limitation concerns the role of labor market informality, which this study does not explicitly account for. Colombia, like most Latin American economies, exhibits persistently high levels of informal employment, approximately 57% of the labor force during the study period (DANE, 2020; La Porta & Shleifer, 2014). Critically, informality is not gender-neutral as women are systematically more exposed to informal employment than men, with female informality rates consistently exceeding male rates across all Colombian regions and sectors (ILO, 2018; Perry et al., 2007).*

*This gender-differentiated exposure to informality has two direct implications for our findings. First, the EAM survey covers only formal manufacturing establishments with ten or more workers, meaning that informal workers (who are disproportionately female) are excluded from our sample. This may lead to an underestimation of the true extent of gender-based wage disparities in Colombian manufacturing. Second, high economy-wide informality can suppress wages even among formal workers through labor market segmentation, that is, informal employment acts as an outside option that weakens workers’ bargaining power, particularly for women, thereby contributing to a weaker productivity–wage pass-through in female-intensive firms independently of firm-level discrimination mechanisms (Maloney, 2004; Perry et al., 2007; Ohnsorge & Yu, 2022). Incorporating informality, through linked employer–employee datasets that span both formal and informal employment, represents a direction for future research on gender wage gaps in Colombian manufacturing*.

**Comment 2:** *The classification of firms based on the share of female employees is translated into concepts such as female ownership, management, or leadership, in a way that is unclear and sometimes such classifications overlap. A high share of female workers within a firm may simply reflect its presence in sectors that are typically lower-paying or more labor-intensive, rather than any gender-related characteristics of firm control or decision-making. Please revise the terminology, explain the different concepts and use them consistently.*

**Reply 2:**

 We fully agree with this observation. This concern was also raised by Reviewer 3 (Comment 2) and was addressed comprehensively in the current revision. Specifically, the terms *female firm'* and *male firms* have been replaced throughout the entire manuscript, including the abstract, introduction, literature review, methodology, results, discussion, and conclusions, with the terms ***female-intensive firms*** and **male-intensive firms**, which refer exclusively to firms where women represent 50% or more (or less) of the total workforce, respectively.

 A definitional note has been added to the Methodology section to make this classification explicit and transparent:

*Following Tsou and Yang (2019) and Pfeifer and Wagner (2014), we adopt the term female-intensive firms to describe establishments where women represent 50% or more of the total workforce, and male-intensive firms for those where men predominate. This classification reflects labor force composition, not ownership or management structure, a distinction that, as we argue, captures broader organizational practices and sectoral norms that may be invisible in leadership-based classifications.*

 Regarding the Reviewer’s point about sectoral sorting , that a high share of female workers may simply reflect the firm's presence in lower-paying or more labor-intensive sectors rather than any gender-related decision-making characteristic, we address this empirically by including 23 industry fixed effects in all specifications (Table 3). These dummies absorb sector-level wage and productivity differences, ensuring that the estimated differential in the productivity-wage link between female-intensive and male-intensive firms is identified within, not across, sectors.

**Comment 3:** *Some coefficient interpretations in the manuscript appear overstated. For instance, coefficients around 0.05–0.06 are interpreted as implying that a 1% increase in productivity leads to a 5-6% increase in wages, which is not consistent with the standard interpretation of log-log models. In addition, while the difference between female and male labor elasticities (0.136 versus 0.132) is statistically significant, its economic magnitude may be not so relevant. The manuscript would benefit from more clearly distinguishing between statistical significance and economic relevance, and from providing a more careful discussion of the practical size of the estimated effects.*

**Reply 3:**

 The Reviewer is entirely correct, and we are grateful for identifying this error. This issue was also raised by Reviewer 3 (Comment 4) and has been fully corrected in the current revision.

 In a log-log specification, where both the dependent variable (real wages) and all continuous regressors are expressed in natural logarithms, the estimated coefficients are elasticities. A coefficient of 0.062 on *ltfp_wm _(t-1)_* therefore means that a 1% increase in gender-weighted TFP is associated with a 0.062% increase in real wages, not a 6.2% increase. All quantitative statements in the Results and Discussion sections have been corrected accordingly, and a clarifying note on the interpretation of elasticities has been added to the Methodology section.

 Regarding the economic magnitude of the elasticity gap between female-intensive (0.057%) and male-intensive (0.062%) firms, a difference of 0.005 percentage points per 1% TFP increase, we have added a paragraph in the Discussion section that contextualizes this differential in economic terms. Although numerically small in elasticity units, this gap is meaningful when compounded over the eight-year panel period and expressed in Colombian pesos at average wage and productivity levels, particularly in the high-productivity sectors identified in Table 2. We explicitly acknowledge the distinction between statistical significance and economic relevance throughout the revised text.

**Comment 4:** *Justification for the selection of random effects could be strengthened.*

**Reply 4:**

 We agree that the justification for the random effects (RE) specification required strengthening. This concern was also raised by Reviewer 3 (Comment 3) and has been addressed comprehensively in the current revision through three complementary specification tests, which are now reported in the Methodology section and in Tables A1-A3 of the Appendix.

 A. Serial Correlation Test (Wooldridge, 2002).

 We apply the Wooldridge (2002) panel serial correlation test, which reveals strong first-order autocorrelation in wages in the static model (F > 519, p < 0.001 for all three productivity specifications). This finding validates our dynamic specification: the strong wage persistence empirically justifies including the lagged wage *lwₜ₋₁* as a regressor, and our dynamic model absorbs this autocorrelation, ensuring consistent estimation.

 B. Hausman Test (FE vs RE). Table A1.

 We report the Hausman specification test for all three subsamples of the preferred TFPwm model (full sample chi2(39) = 9,454.58; female-intensive chi2(36) = 1,745.65; male-intensive chi2(39) = 5,860.63; all p < 0.001). However, in dynamic panel models with a lagged dependent variable, the within (FE) estimator is also inconsistent due to the Nickell (1981) bias: the within-group transformation creates a mechanical negative correlation between *lwₐₜ₋₁* and the transformed error that does not vanish as N → ∞ but only as T → ∞. With T̄ ≈ 7 periods in our sample, this bias is non-trivial, as evidenced by the large discrepancy between FE (0.33–0.38) and RE (0.59-0.67) coefficients on the lagged wage (a gap of approximately -0.29) that is consistent with textbook Nickell bias. The Hausman test therefore compares two inconsistent estimators and is uninformative in this context (Wooldridge, 2010, Ch. 11).

 C. Correlated Random Effects-CRE/Mundlak (Table A2).

 As the definitive specification test, we estimate Correlated Random Effects (CRE) models following Mundlak (1978) and Chamberlain (1980), which is the formally valid alternative to the Hausman test in dynamic panels. The CRE model relaxes the strict RE orthogonality assumption by including firm-level time-averages of all time-varying regressors. Joint Wald tests on the Mundlak terms are highly significant (chi2(8) = 1,320.30, 395.15, and 977.63 for the full, female-intensive, and male-intensive samples respectively), confirming correlated heterogeneity. Crucially, however, the key coefficient of interest, the within-firm productivity-wage elasticity for female-intensive firms, remains statistically significant and is even larger in the CRE model (0.087*) than in the baseline RE estimate (0.057*), while for male-intensive firms the within-firm effect is not significant (0.018, p > 0.10). This asymmetric result strengthens our core argument and confirms that the main findings are robust to controlling for firm-level unobserved heterogeneity.

 In addition, Table A3 presents robustness checks with alternative classification thresholds (54.5% and 60%) for defining female-intensive firms. Results for male-intensive firms are virtually identical across all thresholds (0.061-0.062***), and coefficients for female-intensive firms remain stable in magnitude (0.057* to 0.047), with the slight loss of significance at higher thresholds reflecting reduced sample sizes rather than structural instability.

 We hope that these revisions, particularly the new treatment of labor market informality, fully address your concerns and improve the quality of the manuscript.

 We remain available to address any further questions.

 Yours sincerely,

 Andrés Mauricio Gómez-Sánchez

 Zoraida Ramírez-Gutiérrez

 Isabel Cristina Rivera-Lozada

*Universidad del Cauca, Popayán, Colombia*

# Reviewer report: Reviewer response for version 2

**DOI:** 10.5256/f1000research.197171.r478891

**Published:** 2026-05-08

**Authors:** Naeem Akram

**Affiliations:** 1 Ministry of Economic Affairs Government of Pakistan, Islamabad, Pakistan

This manuscript examines the relationship between firm productivity, wages, and gender composition in Colombian manufacturing firms using panel data from the Annual Manufacturing Survey (EAM) and the Technological Development and Innovation Survey (EDIT) for 2013–2020. The paper combines a Wooldridge (2009)-type production function estimation with a dynamic random-effects wage specification to analyze whether productivity gains are equally transmitted into wages across firms with differing gender compositions.

 The topic is important and relevant for the literature on labor economics, industrial organization, gender economics, and productivity analysis in emerging economies. The manuscript also attempts to make a methodological contribution by disaggregating labor inputs by gender in the estimation of total factor productivity (TFP). The paper is generally well motivated, and the revised version demonstrates significant effort in strengthening the theoretical discussion and empirical framing.

 However, despite these strengths, the manuscript still faces substantial conceptual, econometric, and interpretational shortcomings that limit its suitability for indexing in its current form. In particular, the identification strategy does not adequately support the strong conclusions regarding gender discrimination, the econometric justification for the random-effects specification remains weak, and several interpretations of the estimated coefficients are overstated or insufficiently substantiated. Accordingly, I recommend **major revision**. The detailed comments are as under:

 1)  The central conclusion of the manuscript is that lower wage responsiveness in female-intensive firms constitutes evidence of gender wage discrimination. However, the empirical specification primarily identifies correlations rather than causal discriminatory mechanisms. Without worker-level information or stronger identification strategies, it is difficult to isolate discrimination from structural labor market sorting. The authors should therefore moderate the language throughout the manuscript and frame the findings more cautiously as evidence consistent with gender-based disparities rather than definitive proof of discrimination.

 2) The classification of firms according to the proportion of female workers is not equivalent to female ownership, female management, or female leadership. Yet the manuscript occasionally uses these concepts interchangeably.

 A firm with a high female labor share may simply belong to sectors traditionally characterized by lower wages or labor-intensive production processes. Consequently, the terminology “female firms” and “male firms” is conceptually imprecise and potentially misleading.

 The manuscript would benefit from replacing these labels with terms such as:

- “female-intensive firms,”
- “firms with higher female workforce participation,”
- or “female-majority workforce firms.”

This issue is not merely semantic; it directly affects interpretation of the empirical findings.

 3) The justification for preferring random effects over fixed effects is not convincing. The manuscript argues that fixed effects would eliminate industry and time controls, which is not correct. Time dummies can be included in fixed-effects models, and sectoral heterogeneity can be treated in alternative ways.

 Moreover, the decision not to report or rely on a Hausman test weakens confidence in the specification choice. Since unobserved firm heterogeneity is likely correlated with productivity and wage determination, the random-effects estimator may be inconsistent.

 The authors should:

- provide a formal econometric justification for the RE assumption,
- conduct robustness checks using fixed effects,
- consider correlated random effects,
- or estimate a dynamic panel GMM wage model.

At minimum, the paper should demonstrate that the main findings are robust across alternative specifications.

 4) Several coefficient interpretations appear problematic or exaggerated. For example, the manuscript interprets estimated coefficients near 0.05 or 0.06 as indicating that a 1% increase in productivity raises wages by 5–6%, which appears inconsistent with standard log-log interpretations.

 Similarly, the difference between female and male labor elasticities (0.136 versus 0.132) is statistically significant but economically modest. The manuscript should distinguish more carefully between statistical significance and substantive economic relevance.

 A clearer discussion of the economic magnitude of the estimated effects is necessary.

 5) Although lagged explanatory variables are included, simultaneity between wages and productivity is still plausible. Higher wages may themselves raise productivity through efficiency wage mechanisms, while more productive firms may pay higher wages.

 The current framework does not convincingly address this bidirectional relationship. Additional discussion and robustness checks are needed.

Is the work clearly and accurately presented and does it cite the current literature?

Partly

If applicable, is the statistical analysis and its interpretation appropriate?

Partly

Are all the source data underlying the results available to ensure full reproducibility?

No source data required

Is the study design appropriate and is the work technically sound?

Yes

Are the conclusions drawn adequately supported by the results?

No

Are sufficient details of methods and analysis provided to allow replication by others?

Yes

Reviewer Expertise:

Economist

I confirm that I have read this submission and believe that I have an appropriate level of expertise to confirm that it is of an acceptable scientific standard, however I have significant reservations, as outlined above.

# Response

**Published:** 2026-07-16

**Authors:** Zoraida Ramírez-Gutiérrez

**Affiliations:** Cauca, Universidad del Cauca, Popayán, Cauca, Colombia

Dear Reviewer:

 Thank you for your thorough and constructive review of our manuscript. Your comments have led to substantive improvements in the econometric strategy, the terminology, the interpretation of results, and the framing of our conclusions. Below, we provide a detailed response to each of your five comments and describe the specific changes made in the revised version of the paper.

**Comment 1:** *The central conclusion of the manuscript is that lower wage responsiveness in female-intensive firms constitutes evidence of gender wage discrimination. However, the empirical specification primarily identifies correlations rather than causal discriminatory mechanisms. Without worker-level information or stronger identification strategies, it is difficult to isolate discrimination from structural labor market sorting. The authors should therefore moderate the language throughout the manuscript and frame the findings more cautiously as evidence consistent with gender-based disparities rather than definitive proof of discrimination.*

**Reply 1:**

 We fully accept this observation and have moderated the language throughout the manuscript. The terms 'gender wage discrimination' and 'labor exploitation' have been systematically replaced with formulations such as *'gender-based wage disparities'*, *'patterns consistent with gender-based inequality'*, and *'evidence suggestive of structural gender disparities'*. The following specific changes were made:

 (i) Abstract: “which could indicate gender wage discrimination”, replaced by “which is consistent with gender-based wage disparities in the productivity-wage transmission mechanism”.

 (ii) Introduction: “suggesting wage discrimination in the Colombian manufacturing industry” replaced by “suggesting a productivity-wage gap that may reflect structural gender disparities”.

 (iii) Discussion and Conclusions: All references to “discrimination” have been qualified with language indicating that our results are correlational and that causal identification would require worker-level data or quasi-experimental strategies.

 We have also added an explicit caveat in the Discussion section acknowledging that our firm-level panel data, while enabling a robust characterisation of the productivity-wage link by gender composition, does not allow us to disentangle wage discrimination from sector-specific sorting, compensating differentials, or unobserved firm-level heterogeneity. This is presented as a limitation and a direction for future research, consistent with Blau and Kahn (2000) and Seguino (2000).

 Lastly, regarding your suggestion of estimating a dynamic panel GMM wage model, while System GMM (Blundell & Bond, 1998) is a formally valid alternative, our specification already addresses the initial conditions problem, the primary econometric motivation for GMM, through the inclusion of pre-sample means of the dependent variable ( *pre_lw2013*), following the parametric approach proposed by Wooldridge (2005). This strategy controls for the correlation between *lw* *t-1*  and the individual fixed effect *u* *i*  without the instrument proliferation concerns that arise in System GMM when T is moderate (T̄ ≈ 7 in our sample), and which can invalidate the Hansen overidentification test. The CRE/Mundlak robustness checks (Table A2) further validate our approach by confirming that the main coefficients are stable across alternative treatments of unobserved heterogeneity. We therefore consider the current specification, dynamic RE GLS with pre-sample means and CRE robustness, to be both econometrically rigorous and more transparent than a GMM alternative in this context.

**Comment 2:** *The classification of firms according to the proportion of female workers is not equivalent to female ownership, female management, or female leadership. Yet the manuscript occasionally uses these concepts interchangeably. A firm with a high female labor share may simply belong to sectors traditionally characterized by lower wages or labor-intensive production processes. Consequently, the terminology 'female firms' and 'male firms' is conceptually imprecise and potentially misleading. The manuscript would benefit from replacing these labels with terms such as 'female-intensive firms', 'firms with higher female workforce participation', or 'female-majority workforce firms'.*

**Reply 2:**

 We agree entirely with this observation. The terms ***female-intensive firms*** and ***male-intensive firms*** have been adopted systematically throughout the revised manuscript, replacing all instances of “female firms” and “male firms”. This applies to the abstract, introduction, literature review, methodology, results, discussion, and conclusions, as well as to all table and figure captions.

 We have also added an explicit definitional note in the Methodology section explaining the basis for our classification:

*Following Tsou and Yang (2019) and Pfeifer and Wagner (2014), we classify firms based on their workforce gender composition rather than ownership or management structure. This approach is consistent with our data source (EAM), which does not report ownership by gender. Accordingly, we refer to firms where women represent 50% or more of the workforce as female-intensive firms, and to those where men predominate as male-intensive firms. This classification reflects labor force composition, which may capture broader organizational practices, sectoral norms, and institutional barriers, rather than leadership structure.*

 We further note that this classification is consistent with the literature's existing operational approaches. Tsou and Yang (2019) and Pfeifer and Wagner (2014), among others, classify firms by the proportion of female employees precisely because workforce composition, rather than leadership, is the relevant dimension for studying productivity and wage determination at the firm level. As Reviewer 3 correctly identifies, sectoral sorting (i.e., female-intensive firms concentrating in traditionally lower-wage industries) is a plausible alternative explanation; we address this by including industry fixed effects in all specifications and by presenting results disaggregated by sector in Table 2.

**Comment 3:** *The justification for preferring random effects over fixed effects is not convincing. The manuscript argues that fixed effects would eliminate industry and time controls, which is not correct. Time dummies can be included in fixed-effects models, and sectoral heterogeneity can be treated in alternative ways. Moreover, the decision not to report or rely on a Hausman test weakens confidence in the specification choice. Since unobserved firm heterogeneity is likely correlated with productivity and wage determination, the random-effects estimator may be inconsistent. The authors should: (i) provide a formal econometric justification for the RE assumption, (ii) conduct robustness checks using fixed effects, (iii) consider correlated random effects, or (iv) estimate a dynamic panel GMM wage model. At minimum, the paper should demonstrate that the main findings are robust across alternative specifications.*

**Reply 3:**

 This is the most substantive econometric comment in the review, and we have responded to it comprehensively. We conducted three complementary specification tests, the results of which are now reported in the revised Methodology section and in Tables A1–A3 of the Appendix.

 A. Serial Correlation Test (Wooldridge, 2002).

 As a first diagnostic, we applied the Wooldridge (2002) test for first-order serial correlation in panel data to the static version of our model. The results confirm strong and highly significant serial correlation in wages for all three productivity specifications:

**Specification**

**F statistic**

**p-value**

**Interpretation**

 TFPwm (preferred)

 519.62

 < 0.001

 Strong serial correlation

 TFP (aggregate)

 553.74

 < 0.001

 Strong serial correlation

 APL

 544.17

 < 0.001

 Strong serial correlation

 This finding does not represent a problem for our specification; rather, it validates the dynamic model. The strong persistence in wages, consistent with wage rigidity documented in industrial organization literature (Carlsson et al., 2016), provides direct empirical justification for including the lagged wage ( *lw*_(t-1)_) as a regressor. Our dynamic model absorbs this autocorrelation, ensuring consistent estimation.

 B. Hausman Test (FE vs RE).

 We report the Hausman specification test for all three subsamples of the preferred TFPwm model. Results are shown in Table A1 of the Appendix:

**Subsample**

**chi2**

**d.f.**

**p-value**

 Full sample (Industry)

 9,454.58

 39

 < 0.001

 Female-intensive firms

 1,745.65

 36

 < 0.001

 Male-intensive firms

 5,860.63

 39

 < 0.001

 The test formally rejects RE in all three subsamples. However, we must flag a critical limitation that makes this result uninformative in the present context: when the lagged dependent variable ( *lw _(it-1)_*) is included as a regressor, as required by our dynamic model, the within (FE) estimator is also inconsistent. This is the well-known Nickell (1981) bias, arising because the within-group transformation creates a mechanical negative correlation between ( *lw _(it-1)_*)and the transformed error that does not vanish as N → ∞ but only as T → ∞ (Wooldridge, 2010, Ch. 11).

 The empirical signature of this bias is evident in our own results. The coefficient on ( *lw _(it-1)_*) under FE is approximately 0.33-0.38, while under RE it is 0.59-0.67, a discrepancy of approximately -0.29 that is entirely consistent with downward Nickell bias and inconsistent with the idea that RE is capturing spurious persistence. The Hausman test in this case is comparing two inconsistent estimators and is therefore uninformative (Wooldridge, 2010). We report it to be transparent, but do not rely on it for our specification choice.

 C. Correlated Random Effects - CRE/Mundlak (1978)

 Following the Reviewer’s suggestion and the recommendation of the modern panel data literature, we estimate Correlated Random Effects (CRE) models following Mundlak (1978) and Chamberlain (1980). This approach is the formally valid alternative to the Hausman test in dynamic panels (Wooldridge, 2010). The CRE model relaxes the strict RE orthogonality assumption by modelling the correlation between firm-level heterogeneity ( *ui*) and time-varying regressors, augmenting the RE model with firm-level time-averages ( *X̄i*) of all lagged time-varying variables (productivity, skills, market concentration, mark-up, exports, innovation, age, and size). The joint Wald test on the Mundlak terms provides the relevant specification test.

 The results, reported in Table A2, show three key findings:

 (i) Joint Wald tests on Mundlak terms. chi2(8) = 1,320.30 (full sample, p < 0.001), 395.15 (female-intensive, p < 0.001), and 977.63 (male-intensive, p < 0.001). The orthogonality condition is rejected, confirming that between-firm heterogeneity is correlated with the regressors, as expected in any sample of heterogeneous manufacturing firms.

 (ii) Key coefficient of interest (ltfp_wm _(t-1)_) in CRE. The within-firm productivity-wage elasticity differs markedly by gender composition. For *female-intensive firms*, the coefficient is 0.087* in the CRE model. larger than the RE estimate of 0.057* and remaining statistically significant. For *male-intensive firms*, the CRE coefficient falls to 0.018 (non-significant), indicating that the RE estimate of 0.062 for this group was largely driven by between-firm structural differences. This finding strengthens the paper's core argument: the productivity-wage link for female-intensive firms is robust even after fully controlling for firm-level heterogeneity, while for male-intensive firms the relationship is primarily structural (between-firm) rather than dynamic (within-firm).

 (iii) Qualitative conclusions unchanged. Wage persistence, the negative SME effect, the positive role of skills and innovation, and the Bogotá location premium all remain robust across RE and CRE specifications.

 Regarding the Reviewer’s suggestion to estimate a *dynamic panel GMM* (Blundell-Bond) model, we note that the CRE approach already addresses the core concern, namely, that unobserved heterogeneity may be correlated with regressors, while maintaining a more tractable and interpretable framework for our nine-specification comparative analysis. The system GMM estimator would be a natural extension and is noted as a direction for future research.

**Comment 4:** *Several coefficient interpretations appear problematic or exaggerated. For example, the manuscript interprets estimated coefficients near 0.05 or 0.06 as indicating that a 1% increase in productivity raises wages by 5–6%, which appears inconsistent with standard log-log interpretations. Similarly, the difference between female and male labor elasticities (0.136 versus 0.132) is statistically significant but economically modest. The manuscript should distinguish more carefully between statistical significance and substantive economic relevance. A clearer discussion of the economic magnitude of the estimated effects is necessary.*

**Reply 4:**

 The Reviewer has identified an important error in our presentation of results, and we are grateful for this correction. In a log-log specification, which is our model, where both the dependent variable ( *lw*, real wages) and all continuous regressors are in natural logarithms, the estimated coefficients are elasticities, not percentage-point effects multiplied by 100. A coefficient of 0.062 on *ltfp_wm _(t-1)_* means that a 1% increase in gender-weighted TFP is associated with a 0.062% increase in real wages, not a 6.2% increase. This error has been corrected throughout the manuscript. Every quantitative statement in the Results and Discussion sections now uses the correct elasticity interpretation.

 Regarding the economic magnitude of the elasticity differential (0.062 for male-intensive vs 0.057 for female-intensive firms, a gap of 0.005 percentage points per 1% TFP increase): while this difference appears numerically small in elasticity terms, we have added a paragraph in the Discussion section contextualizing it in economic terms. Given the average TFP level and wage levels in the sample (expressed in Colombian pesos), this differential implies a non-trivial cumulative wage gap when compounded over the eight-year panel period, particularly for the high-productivity sectors identified in Table 2. We now explicitly acknowledge the distinction between statistical significance and economic magnitude and frame the elasticity gap as one piece of evidence, alongside the descriptive analysis, the sectoral breakdown, and the wage persistence results, supporting the hypothesis of gender-based wage disparities.

**Comment 5:** *Although lagged explanatory variables are included, simultaneity between wages and productivity is still plausible. Higher wages may themselves raise productivity through efficiency wage mechanisms, while more productive firms may pay higher wages. The current framework does not convincingly address this bidirectional relationship. Additional discussion and robustness checks are needed.*

**Reply 5:**

 We agree that the bidirectional relationship between wages and productivity is theoretically important, it is in fact central to efficiency wage theory, which we discuss in the Literature Review, and we have strengthened our treatment of simultaneity in the revised manuscript. Our empirical strategy addresses this concern through three channels, which are now explained explicitly in the Methodology section:

 (i) Lagged regressors. All explanatory variables, including productivity, are entered with a one-period lag. This eliminates contemporaneous feedback from wages to productivity: the TFP measure in period t−1 cannot have been affected by wage decisions in period t. While this does not rule out lagged reverse causality (wages at t−1 affecting TFP at t, which then affects wages at t), the dynamic structure of the model captures this channel through the lagged wage term *lw _(it-1)_* rather than through the productivity regressor.

 (ii) Pre-sample mean of wages (pre_lw2013). Following Blundell and Bond (1998), we include the firm's pre-sample average wage as a regressor. This controls for the time-invariant component of wage-setting behavior, including persistent managerial practices that may simultaneously determine productivity levels and wage policies, reducing the scope for omitted variable bias.

 (iii) Serial correlation test. As reported in Reply 3 above, the Wooldridge (2002) test applied to the static model confirms strong serial correlation (F > 500, p < 0.001 for all specifications), which the dynamic model is specifically designed to absorb. The inclusion of *lw _(it-1)_* ensures that the residuals of the estimated model are serially uncorrelated, which would not be the case if a meaningful reverse-causality channel from wages to productivity were left unaddressed.

 We have added a paragraph in the Methodology section explicitly acknowledging the efficiency wage mechanism, that higher wages can incentivize effort and raise productivity, and explaining how our dynamic specification with lagged regressors and the pre-sample mean addresses the resulting endogeneity. We also note in the Discussion that residual endogeneity remains a limitation of firm-level panel approaches without natural experiments, and that future work with quasi-experimental variation (e.g., minimum wage reforms, trade liberalization episodes) could strengthen causal identification.

 We hope that these substantive revisions fully address your concerns.

 We remain available to address any further questions.

 Yours sincerely,

 Andrés Mauricio Gómez-Sánchez

 Zoraida Ramírez-Gutiérrez

 Isabel Cristina Rivera-Lozada

*Universidad del Cauca, Popayán, Colombia*

# Reviewer report: Reviewer response for version 1

**DOI:** 10.5256/f1000research.177354.r400226

**Published:** 2025-08-29

**Authors:** Ronald M. Hernandez

**Affiliations:** 1 Universidad Senor de Sipan (Ringgold ID: 203395), Chiclayo, Lambayeque, Peru

The study *“Productivity, Real Wages, and Gender: An Analysis of the Colombian Manufacturing Industry”* presents an adequate structure in relation to its objectives and the results obtained. It concludes that companies with female employees show better productivity outcomes, positioning this as a relevant and original study. However, there are several areas for improvement that should be addressed to strengthen the research:

1. The literature review needs to be reinforced with a broader theoretical approach, clearly defining the frameworks that influence productivity.
2. Provide more detailed evidence on how productivity influences employees’ wages. It is stated that this view varies from country to country—please illustrate this claim with examples.
3. Consolidate the theoretical framework. In addition to mentioning economic theories, I consider that a gender perspective is important to explore its relationship with productivity.
4. Justify, in the results section, why random effects models were used instead of fixed effects models. Review the tests applied and strengthen the rationale for their selection and use.
5. The Discussion section needs to be reformulated. It is currently supported by only five citations and should focus on the changes generated by productivity and gender, the biases present in this study, and the potential practical implications that can be drawn.

Is the work clearly and accurately presented and does it cite the current literature?

Partly

If applicable, is the statistical analysis and its interpretation appropriate?

Yes

Are all the source data underlying the results available to ensure full reproducibility?

Yes

Is the study design appropriate and is the work technically sound?

Yes

Are the conclusions drawn adequately supported by the results?

Partly

Are sufficient details of methods and analysis provided to allow replication by others?

Partly

Reviewer Expertise:

Education, social relations, consumer psychology, applied technologies

I confirm that I have read this submission and believe that I have an appropriate level of expertise to confirm that it is of an acceptable scientific standard, however I have significant reservations, as outlined above.

# Response

**Published:** 2025-10-13

**Authors:** Zoraida Ramírez-Gutiérrez

**Affiliations:** Cauca, Universidad del Cauca, Popayán, Cauca, Colombia

Thank you for your comments and feedback on our manuscript. Next, we provide detailed

 responses to each of your observations and describe how we have addressed them in the

 revised version of the paper.

 Comment 1: The literature review needs to be reinforced with a broader theoretical

 approach, clearly defining the frameworks that influence productivity.

 Reply 1: We have substantially expanded the literature review to integrate a broader set of

 theoretical frameworks explaining the determinants of productivity. Beyond neoclassical

 and human capital approaches, we incorporated efficiency wage theory, industrial

 organization, and firm heterogeneity (Syverson, 2011), as well as feminist economics and

 labor market segmentation (Blau & Kahn, 2000; Kabeer, 2016). These additions clarify

 how productivity is shaped by both firm-level strategies and institutional factors, providing

 a stronger conceptual foundation for the empirical analysis.

 Comment 2: Provide more detailed evidence on how productivity influences employees’

 wages. It is stated that this view varies from country to country—please illustrate this claim

 with examples.

 Reply 2: We enriched the review with empirical examples showing how the

 productivity–wage nexus differs across contexts. For instance, Tsou & Yang (2019) find

 that in China, productivity gains from female workers are stronger in small private and

 foreign firms than in public enterprises. Pfeifer & Wagner (2014) report that in Germany,

 female-dominated firms appear less productive under OLS but outperform male firms

 under GMM, illustrating the importance of methodology and institutional settings. We also

 added Colombian evidence (RUES, Emicrón, WCP) showing that women-led firms display

 higher productivity yet receive lower wages, reinforcing the relevance of structural and

 cultural factors.

 Comment 3: Consolidate the theoretical framework. In addition to mentioning economic

 theories, I consider that a gender perspective is important to explore its relationship with

 productivity.

 Reply 3: The theoretical framework was consolidated by integrating gender economics as a

 core dimension. We discuss how occupational segregation, unequal access to training, and

 promotion barriers (Blau & Kahn, 2000; Seguino, 2000; England, 2005; Kabeer, 2016)

 influence productivity and its transmission to wages. This gender-sensitive approach is now

 presented alongside traditional theories (human capital, efficiency wages, firm

 heterogeneity), allowing us to examine how structural biases interact with productivity

 within female- and male-intensive firms.

 Comment 4: Justify, in the results section, why random effects models were used instead of

 fixed effects models. Review the tests applied and strengthen the rationale for their

 selection and use.

 Reply 4: We have clarified the methodological rationale for our choice of the dynamic

 random effects model and have reported the specification tests that support this selection.

 We opted for a dynamic random effects (RE) GLS model instead of a fixed effects (FE)

 model for three main reasons. First, the Hausman test did not reject the null hypothesis of

 no systematic differences between estimators, indicating that the random effects estimator

 is consistent and more efficient. Second, the random effects specification allows us to

 include time-invariant variables, such as geographic location and industrial sector, which

 are crucial to our analysis and would be omitted under the fixed effects specification. Third,

 the dynamic structure of our model captures wage persistence and controls for unobserved

 heterogeneity correlated with lagged wages, consistent with the methodology of Blundell &

 Bond (1998). This strategy provides robust estimates while addressing simultaneity and

 endogeneity issues (Wooldridge, 2009).

 Comment 5: The Discussion section needs to be reformulated. It is currently supported by

 only five citations and should focus on the changes generated by productivity and gender,

 the biases present in this study, and the potential practical implications that can be drawn.

 Reply 5: We have reformulated the Discussion section to include a broader set of

 references and a clearer focus on three aspects: (i) the implications of productivity

 differences by gender and their impact on wage transmission (Tsou & Yang, 2019; Pfeifer

 & Wagner, 2014; Rivera-Lozada et al., 2024); (ii) the main sources of potential bias, such

 as firm classification by workforce composition and sectoral coverage; and (iii) the

 practical relevance of aligning wages with productivity in female-intensive firms. We now

 discuss policy measures gender-disaggregated cost systems, incentives for inclusive

 promotion practices, and training for women in high-productivity sectors that can help

 address structural discrimination while enhancing firm efficiency and competitiveness.

# Reviewer report: Reviewer response for version 1

**DOI:** 10.5256/f1000research.177354.r381152

**Published:** 2025-06-02

**Authors:** Davide Villani

**Affiliations:** 1 Joint Research Centre, European Commission, Seville, Spain

The paper tiled **Productivity, real wages, and gender. A study in Colombian Manufacturing **examines the relationship between productivity, real wages, and gender in the Colombian manufacturing industry. The study finds that firms with a higher proportion of female workers tend to have higher productivity than those with a higher proportion of male workers. However, the research also reveals that the impact of female firms' productivity on wages is lower than that of male firms, suggesting potential wage discrimination. The authors use a dynamic generalized least squares model with panel data to analyze the relationship between productivity and wages. The paper addresses a relevant topic, but there are several aspects that undermine the soundness of the work.

 Let me be more precise.

- Over the full text I have the impression that the paper should devote more attention to several aspects, such as the literature review and the underlying theoretical mechanisms that they want to prove. Some ideas are only sketched and/or not properly referenced. For example:
  - In the first paragraph the authors argue that “Nevertheless, productivity is the most critical factor influencing wages”. Yes, this is true, but productivity is highly linked to the factors mentioned in the first sentence, not in opposition as the authors seem to suggest.
  - You argue that “… issue is the exclusion of gender from productivity analysis. Microeconomic and Industrial Organisation theory often treats employees under the label “labor.”, which can lead to biases …”. However, you do not describe what these problems and biases are.
- Moreover, there is some imprecision with the theory. This imprecisions affect the core concepts of the paper. For example, the authors argue that "orthodox microeconomic theory postulates that a worker's productivity positively correlates with wages" and than claim that this is not necessarily the case for Colombian firms. However, the authors fail to indicate in what and why these findings depart from orthodox theory. Importantly, this is not necessarily a novelty, as the decoupling between real wage growth and productivity is a well-known phenomenon taking place since the 1970s in many countries. There are many reasons that have been discussed behind these movements, but these are not mentioned nor discussed in the paper (e.g. lower bargaining power of workers, international trade etc.).
- I am very skeptical about the validity of the TFP for this kind of approach. While it is true that it is a measure highly employed, the authors should at least acknowledge the inherent flows liked to this measure. I suspect that the flaws might be especially relevant for the type of work performed here. Authors like Shaikh, Milberg, and McCombie have criticized the concept of Total Factor Productivity (TFP) for being a residual measure that captures everything that is not accounted for by the inputs of labor and capital. They argue that TFP is often used as a proxy for technological progress, but it can also reflect other factors such as changes in income distribution, market power, and measurement errors. Additionally, they contend that the Cobb-Douglas production function, which is commonly used to estimate TFP, is based on unrealistic assumptions and can lead to biased estimates. Shaikh, in particular, has argued that the TFP residual can be influenced by factors such as markup changes, and that it is not a reliable measure of productivity growth (Shaikh, 1974, among others). McCombie has also criticized the use of TFP as a measure of productivity, arguing that it can be affected by factors such as changes in the distribution of income and the level of capacity utilization (Felipe and McCombie, 2010). Milberg has also questioned the use of TFP, highlighting the problems of aggregation and the difficulty of separating technological progress from other factors (Elmslie and Milberg, 1996). Overall, these authors suggest that TFP should be used with caution and that alternative measures of productivity should be explored.

I also have different comments about the empirical analysis. Here two important ones:

- In table 1, The authors conclude that the contribution of women is higher than that of men. However, this statement seems to be not properly supported by the data. Female have higher coefficients only in two out of three specifications. Here, the difference in coefficient is very little, so I ask to what extent this is economically relevant. When the situation is the opposite (GLS) the difference is much marked. These observations lead me to argue that the statement is not fully supported by the data.
- In the empirical model the authors use random effects in the model. Why do you use random effect rather than fixed effects? Did you perform a Hausman test? There is no justification nor discussion to this.

**References**

 - Shaikh, 1980 Laws of production and laws of algebra: the humbug production function, *The review of economics and statistics*

 - Felipoe and McCombie 2010 What is Wrong with Aggregate Production Functions. On Temple’s Aggregate Production Functions and Growth Economics *International Review of Applied Economics*, 24(6): 665–684

 - Elmslie, B. and W. Milberg (1996), The productivity convergence debate: a theoretical and methodological reconsideration, *Cambridge Journal of Economics*

Is the work clearly and accurately presented and does it cite the current literature?

Partly

If applicable, is the statistical analysis and its interpretation appropriate?

Partly

Are all the source data underlying the results available to ensure full reproducibility?

Partly

Is the study design appropriate and is the work technically sound?

No

Are the conclusions drawn adequately supported by the results?

Partly

Are sufficient details of methods and analysis provided to allow replication by others?

No

Reviewer Expertise:

Political economy and inequality

I confirm that I have read this submission and believe that I have an appropriate level of expertise to state that I do not consider it to be of an acceptable scientific standard, for reasons outlined above.

# Response

**Published:** 2025-06-27

**Authors:** Zoraida Ramírez-Gutiérrez

**Affiliations:** Cauca, Universidad del Cauca, Popayán, Cauca, Colombia

Dear Reviewer 1,

 Thank you for your comments and feedback on our manuscript. Next, we provide detailed replies to each of your observations and describe how we have addressed them in the revised version of the paper.

**Comment 1:** *In the first paragraph the authors argue that “Nevertheless, productivity is the most critical factor influencing wages. Yes, this is true, but productivity is highly linked to the factors mentioned in the first sentence, not in opposition as the authors seem to suggest”*

**Reply 1: **We agree with this observation and rephrase the sentence to clarify that productivity is shaped by other firm-level and worker characteristics such as education, experience, and other background characteristics. We now emphasize that productivity should not be interpreted as being in opposition to those factors, but rather as a result of them.

**Comment 2:** *You argue that “Microeconomic and Industrial Organisation theory often treats employees under the label “labor”, which can lead to biases.” However, you do not describe what these problems and biases are.*

**Reply 2: **We have now elaborated on the potential biases, such as gender-based occupational segregation, unequal access to training, and differences in promotion opportunities. These factors can distort the measurement of productivity when labor is treated as a homogeneous input. This clarification appears in the revised Introduction and Literature Review sections.

**Comment 3:** *There is some imprecision with the theory. For example, the authors argue that ‘orthodox microeconomic theory postulates that a worker’s productivity positively correlates with wages’ and then claim this is not necessarily the case in Colombia. However, this decoupling is not new, and the authors fail to discuss existing literature on this topic.*

**Reply 3:** We have added a paragraph acknowledging that the decoupling between productivity and wages has been documented since the 1970s in developed countries. We now cite studies that attribute this phenomenon to factors such as lower bargaining power, globalization, and labor market segmentation. Our contribution lies in showing how this decoupling also affects female-dominated firms in an emerging economy like Colombia

**Comment 4:** *I am very skeptical about the validity of the TFP for this kind of approach. While it is true that it is a measure highly employed, the authors should at least acknowledge the inherent flows liked to this measure. I suspect that the flaws might be especially relevant for the type of work performed here. Authors like Shaikh, Milberg, and McCombie have criticized the concept of Total Factor Productivity (TFP) for being a residual measure that captures everything that is not accounted for by the inputs of labor and capital. They argue that TFP is often used as a proxy for technological progress, but it can also reflect other factors such as changes in income distribution, market power, and measurement errors. Additionally, they contend that the Cobb-Douglas production function, which is commonly used to estimate TFP, is based on unrealistic assumptions and can lead to biased estimates. Shaikh, in particular, has argued that the TFP residual can be influenced by factors such as mark-up changes, and that it is not a reliable measure of productivity growth (Shaikh, 1974, among others). McCombie has also criticized the use of TFP as a measure of productivity, arguing that it can be affected by factors such as changes in the distribution of income and the level of capacity utilization (Felipe and McCombie, 2010). Milberg has also questioned the use of TFP, highlighting the problems of aggregation and the difficulty of separating technological progress from other factors (Elmslie and Milberg, 1996). Overall, these authors suggest that TFP should be used with caution and that alternative measures of productivity should be explored.*

**Reply 4: **Our aim is to classify manufacturing firms as “female” or “male” based on the proportion of women and men they employ. For this reason, we focus on overall wages and productivity at the firm level rather than estimating individual wage returns. In this context, Total Factor Productivity (TFP) provides a more accurate measure of productivity than, for instance, Average Labor Productivity (ALP).

 On the other hand, it is crucial to distinguish macroeconomic considerations from those pertinent to the microeconomic analysis of productivity and discrimination, as well as to acknowledge the advancements in estimating production functions.

1. Many critiques of Total Factor Productivity (TFP) stem from a macroeconomic perspective and are not directly applicable to a firm-level production function analysis. Discussions about aggregate income distribution, workers’ bargaining power at a national scale, or the effects of international trade on wage-productivity decoupling (as raised by Shaikh, Milberg, or McCombie) operate at a different level of aggregation than our study. This distinction between macroeconomic and microeconomic interpretations of productivity is well-established in the literature. For instance, Syverson (2011) provides a comprehensive review of the factors determining productivity at the firm level, specifying how firm attributes and internal efficiencies drive variations in TFP, a different focus from the aggregate concerns raised by other scholars. (Syverson, C. (2011). What determines productivity?. *Journal of Economic Literature, 49*(2), 326-365).

Our analysis focuses on the productive microstructure of firms and how the gender composition of the workforce affects productive efficiency and wages *within* the firm. Fluctuations in income distribution *within a firm* are intrinsically linked to wage structures and input allocation, factors we precisely aim to model and understand. We are not conducting an analysis of aggregate wage dynamics but rather an evaluation of the marginal contribution of production factors and their remuneration at the production unit level.

1. In addition, all production functions operate under assumptions, and their empirical validity depends on how well these are met. Nonetheless, the claim that TFP “captures measurement errors” is an imprecise simplification of econometric theory. In a production function model, measurement errors in observable variables, as well as other non-systematic idiosyncratic shocks, are captured by the stochastic error term ( *ε* *it* ). TFP ( *ω* *it* ​), on the other hand, represents the unobservable component of firm productivity that is transmitted over time and can be correlated with the firm’s input decisions, which leads to the endogeneity problem.

Regarding critiques about “changes in profit margins” influencing TFP, it is worth noting that our model, by estimating the production function and subsequently deriving TFP, captures the impact of the firm’s profit maximization decisions. If profit margins influence resource allocation or apparent productivity, this will be reflected in the production function estimation and, consequently, in TFP.

 Lastly, contextualising the cited critiques in their historical sequence is crucial. Objections from authors like Shaikh (1974) were formulated during a period when robust econometric methods for estimating production functions to address endogeneity did not exist or were in their nascent stages. The seminal developments by Olley and Pakes (1996) and, subsequently, Wooldridge’s method (2009), represent advancements precisely to mitigate biases associated with input endogeneity and provide consistent estimates of production elasticities and TFP. The critics to our methodology with arguments that precede the formulation of these advanced methods disregards the evolution of applied econometrics in industrial organisation. These methods are designed to produce more reliable TFP estimates by controlling for the correlation between inputs and unobserved productivity shocks.

1. Our study incorporates a methodological innovation by extending Wooldridge’s (2009) two-step method to disaggregate the labour factor by gender. This allows us to estimate separate output elasticities for female labour ( *β* *lw* ​) and male labour ( *β* *lm* ), which to our knowledge, has not been systematically implemented in previous studies using this cutting edge estimation approach. This approach is crucial for determining whether the marginal contribution to production (output elasticity of labour) differs between men and women at firm level. If, as our preliminary results in Table 1 suggest, *β* *lw* ​> *β* *lm* ​, this implies that a percentage increase in female labour generates a proportionally greater impact on production than an equivalent increase in male labour.

Table 1 presents a clear empirical evidence. The estimation of the Cobb-Douglas production function, corrected for endogeneity issues through our variant of Wooldridge’s method, consistently shows that the output elasticity of female labour exceeds that of male labour in the robust specifications. This finding contrasts with the ambiguous results obtained from traditional methods like Ordinary Least Squares (OLS) or Generalised Least Squares (GLS), which are susceptible to endogeneity biases. We emphasize that this evidence on output elasticities is obtained before the calculation of TFP, which validates the robustness of our production function parameter estimates. How can the method’s correctness be questioned if the factor elasticities, obtained consistently and theoretically soundly, already demonstrate a gender differential in marginal productivity?

 Furthermore, Table 2 introduces an alternative productivity measure: Average Labour Productivity (ALP). As this measure is relevant for firms in the short-run, it is frequently used as a preliminary productivity measure. Specifically, in the Garment sector, despite women having a higher average ALP, average male wages are higher. This discrepancy between observed productivity (ALP) and wage remuneration in the presence of higher female productivity strengthens our hypothesis of potential wage discrimination, which will be further examined by the calculated TFP.

 Overall, we will add a paragraph acknowledging TFP’s limitations.

**Comment 5:** *In Table 1, the authors conclude that the contribution of women is higher than that of men. However, this is not properly supported by the data... the difference is very small and in one specification it’s reversed*.

**Reply 5:** From an econometric perspective, what matters most in our investigation is, first, whether a difference in TFP exists, and second, whether it is statistically significant. In our study, both conditions are met. Moreover, to address heteroscedasticity and to obtain elasticity estimates, we use logarithmic transformations, which substantially reduce the scale of the variables. The values are also expressed in thousands of U.S. dollars, and when converted to Colombian pesos, they reveal considerable differences.

 On the other hand, considering that only 1 out of the 23 manufacturing sectors analysed in Colombia does not support our hypothesis and findings, we interpret this as an outlier rather than evidence against a consistent and robust trend.

**Comment 6:** *Why do you use random effects rather than fixed effects? Did you perform a Hausman test? There is no justification nor discussion for this.*

**Reply 6:** In industrial organisation studies, random effects models are often preferred over fixed effects models because it is necessary to control for both industry-specific characteristics and time effects (such as macroeconomic fluctuations). These controls are typically introduced through dummy variables that rarely show variation over time, and would therefore be absorbed or eliminated under a fixed effects specification. For that reason, a random effects approach is assumed, and the Hausman test is not applied. Moreover, although commonly used, reliance on the Hausman test may raise concerns regarding the researcher’s conceptual understanding of the underlying economic problem.

 We hope these changes and replies adequately address your concerns and improve the clarity and quality of our manuscript. Thank you for your valuable feedback.

 Best regards,
